# Cell populations simulated in silico within *SimulCell* accurately reproduce the behaviour of experimental cell cultures

**DOI:** 10.1038/s41540-025-00518-w

**Published:** 2025-05-17

**Authors:** Elvira Toscano, Elena Cimmino, Angelo Boccia, Leandra Sepe, Giovanni Paolella

**Affiliations:** 1 CEINGE Biotecnologie Avanzate Franco Salvatore, Naples, Italy; 2 Department of Molecular Medicine and Medical Biotechnology, Università Degli Studi di Napoli “Federico II”, Naples, Italy

**Keywords:** Biotechnology, Cell biology, Computational biology and bioinformatics

## Abstract

In silico simulations are used to understand cell behaviour by means of different approaches and tools, which range from reproducing average population trends to building lattice-based models to, more recently, creating populations of individual cell agents whose mass, volume and morphology behave according to more or less precise rules and models. In this work, a new agent-based simulator, *SimulCell*, was conceived, developed and used to predict the behaviour of eukaryotic cell cultures while growing attached to a flat surface. The system, starting from time-lapse microscopy experiments, uses growth, proliferation and migration models to create synthetic populations closely resembling original cultures. Support for cell-cell and cell-environment interaction makes cell agents able to react to changes in medium composition and other events, such as physical damage or chemical modifications occurring in the culture plate. The simulator is accessible through a web application and generates data that can be shown as tables and graphs or exported for further analyses.

## Introduction

Cell cultures are widely used in biomedical research and play an important role in different research fields, such as biochemistry, cell and molecular biology, pharmacology, just to name a few. Analysis of cell cultures often uses qualitative models to interpret cell behaviour and/or intracellular molecular pathways. Dynamic microscopy and quantitative data analysis have been used to obtain parameters descriptive of specific aspects of cell morphology and functionality and to understand how the behaviour of a cell population changes over time or reacts to perturbations^1–5^. Quantitative models and simulations have often provided the opportunity to test hypotheses, make predictions trying to fill gaps in knowledge, set up experimental procedures or evaluate conditions that would be difficult to obtain with in vitro cell cultures^6^. In silico simulations, by integrating information from various sources, help bridge the gap between concepts, models and experimental data and may provide a better view of cell behaviour^7^. In recent years, they have been used to analyse problems in population genetics^8^, to study neuronal activity^9–11^, to investigate cell dynamics in the colonic crypt^12,13^ or to model liver regeneration^14,15^, among others. Understanding the effects of biological perturbations can suggest ways to intervene and reestablish proper cell function in diseases such as cancer or in autoimmune disorders^16–21^.

Tools and procedures aimed to simulate cell behaviour range from cell mass simulation, where a generic cell represents the behaviour of a whole population^22–24^, to tools where cells are represented by one or more lattice elements, among which a relevant example is CompuCell3D^25,26^ which uses Glazier-Graner-Hogeweg (GGH) models based on Cellular Potts Method (CPM) to simulate cell behaviour and dynamics at single-cell resolution in the context of cell organisation into tissues. CompuCell3D has been used to variously describe cell growth, division, differentiation, death, motion and contact with neighbouring cells^27–29^. Its combination with a C++ library for two-dimensional CPM led to the development of a comprehensive simulation environment, the Tissue Simulation Toolkit^30,31^, which has been widely used for the implementation of in silico models for studying cell migration and morphology in different tissue contexts^32–35^. More recently, simulation systems have been shifting towards models based on cell agents where mass, volume and morphology behave according to rules, thus allowing additional flexibility as each cell can depend on its neighbours and the surrounding environment. Among these, *CellSys* is a modular software tool for off-lattice simulation of cell growth and organisation in multi-cellular systems where each cell is modelled by an isotropic, elastic and adhesive sphere capable of migration, growth and division^36^*. PhysiCell* is another open source agent-based simulator able to handle many interacting cells in dynamic tissue microenvironments. It was originally developed for studying cancer cells and includes a standard library of customisable sub-models for cell volume changes, cycle progression, apoptosis and necrosis, cell-cell interaction and motility^37,38^. Some simulation tools rely on *MaBoSS* to enable probabilistic simulations of cellular networks, where molecules are boolean variables^24,39^. *UPMaBoSS*^40^ produces dynamic populations of interacting cells, associating cell fate decision, division and death, while *PhysiBoSS* simulates spherical cells that can grow/shrink, divide, move, die while interacting with their environment or other cells^41,42^.

Some simulations only model specific aspects of cell behaviour, such as cell fate decisions related to proliferation, death or differentiation. Fouliard et al.^43^ for example, represent the evolution of cultured stem cells by modelling a heterogeneous cell population, in which cell cycle alternates between a quiescence phase and a proliferation phase: local growth medium composition influences the transition probability between cycle phases and cell differentiation^43^. Similarly, Altinok et al.^44^ used a simple cell cycle model, where cells switch between phases, divide into daughter cells or exit cycle because of death, to study how cell cycle dynamics is affected by factors as phase duration, population size and initial conditions^44^. A purely mechanical approach was used by Taylor et al.^45^ to integrate cell motion and cell-cell interaction in 2-dimensional cultures. Simulated cells move by combining random and persistent components while interactions are introduced as steric repulsion and/or as cell-cell attraction by transient links between cells^45^. Movement paths may also be produced within *CelltrackR* by combining a range of movement features, although no support is included for interactions between cells or reaction to other stimuli^46^.

In this work, a novel simulation system and tool, *SimulCell*, is proposed, based on cell agents which integrate movement, growth and other features to produce synthetic cell populations which can closely reproduce the behaviour of experimental ones. Here, simulated cell agents control growth, survival and replication as well as cell cycle transitions, considering volume changes and external stimuli, such as local cell confluence and the presence of growth factors or other drugs in the medium. Movement simulation builds on a previously developed motion model, representing the movement of experimental cell populations in terms of random, persistence and bias components^47^, and adds the ability to modify movement according to cell-cell repulsion, attractant molecules, cell health and metabolism, attachment state and cell cycle. The system is accessible through a web application which allows to run simulations and evaluate results in numerical and graphic format.

## Results

### Growth, proliferation and survival of single cell agents in simulated populations

**Fig. 1:**
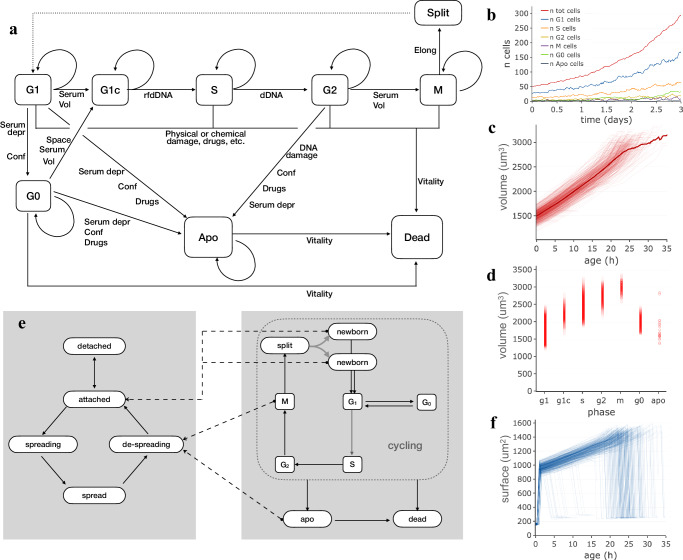
Simulation of cell cycle progression, survival and proliferation. Markov models used in simulating cell attachment and proliferation and cell population produced through them. **a** Schema of states and transitions used to simulate phase changes and cell replication. **b** Growth of a simulated population with average duplication time of 24 h in which cells are duplicated according to the Markov chain based cell cycle model; the different lines report the number of cells in the different cycle phases over time. **c** Cell volume plotted as a function of cell age. Each line corresponds to one of the simulated cells in the population. The thicker curve represents the average volume. **d** Cell volume values in different cycle phases; circles correspond to individual cells at a given time. **e** The succession of states used to model attachment and spreading to the plate substratum. Dashed arrows indicate phase changes in the cell cycle which produce forced changes in attachment state. **f** Cell surface plotted, separately for each cell, as a function of cell age.

Cell proliferation is possibly the most visible feature of a growing cell population and, during every experiment, cells grow, die or progress in the cell cycle until they eventually reach mitosis, split and give rise to daughter cells. In an attempt to produce synthetic cell populations composed of independent cell agents interacting with each other, a simple but flexible procedure was setup, in which cell cycle transitions and mitosis depend on a combination of factors which take into account volumetric growth, extracellular signalling and DNA replication. The developed procedure uses a Markov chain approach to model cell cycle progression as a succession of states, broadly related to cycle phases, where cells move from one state to the next according to probabilities which depend on several internal or external factors (Fig. 1a). In the model, during G_1_ phase cells may pass a checkpoint and move to a G_1c_ state where cells are committed to S phase in dependence of current volume and extracellular signalling provided by serum or other factors. Similarly, G_2_-M phase transition depends on a combination of current cell volume and serum type and concentration. Entering S phase depends on genomic DNA being ready for duplication (*rfdDNA*), while complete DNA duplication is needed to move to G_2_ phase. During M phase, nucleus breakdown and cell elongation result into cell duplication. In addition to the “main loop” phases, G_1_ cells can temporarily exit the cycle by entering G_0_ and optionally reenter it by moving into G_1c_, influenced by cell volume, local cell crowding and/or serum type and concentration. When cells are highly confluent or low serum concentration persists, they have a higher probability to undergo apoptosis which then leads to decrease in vitality and death. Cell death may also be the result of cell damage by physical events, like a scratch, or drug addition to the medium. Damaged cells can recover from damage or undergo death according to the damage level. Volumetric cell growth is simulated by an exponential model in which, during each time interval (*Δt*), cell volume increases according to

1 $${V}_{t}={V}_{c}\cdot{e}^{\alpha \Delta t}$$

where, *V*_*c*_ is the current volume and *α* is the growth rate, which in turn depends on cell type and cycle phase and varies over time and among cells, which may grow faster or slower than the population average. The described procedure works effectively with time intervals ranging between a few minutes and a few hours, according to the need. An example population, simulated with this model and followed for 3 days at 20 min time interval, is shown in Fig. 1b–d. The number of cells (Fig. 1b) exponentially increases over time, with an average replication time of about 24 h and a small portion of cells undergoing apoptosis and death. The accuracy of the replication time obtained by simulating cell cycle in this way was tested by comparing requested versus calculated replication time (average cell age at mitosis) in a panel of simulated populations (Supplementary Table 1): for all of them, the two values turned out to be always very close, even with a broad range of replication times (20–32 h); the apparent duplication time of the same populations is also coherent with the expectations as it is necessarily larger due to the fraction of cells which die or spend time in G_0_ before undergoing apoptosis.

Cell cycle phase composition, as should be expected, reflects phase durations and is maintained over time: most cells (50–60%) are in G_1_, 20–30% of cells are in S phase, while smaller fractions of cells are in the other phases (Fig. 1b). Cell volume, plotted as a function of age separately for each cell (Fig. 1c), shows an overall increase pattern although, within the same population, curves differ for different cells as internal state and external factors play a role in generating variability between cells. Cell volume changes during the cell cycle (Fig. 1d), progressively increasing from G_1_ to M phase. Cell numbers and volumes, calculated according to this model, are coherent with known doubling times and not far from growth patterns experimentally observed in different cell cycle phases^48–52^.

Cells growing on a culture dish change shape and attachment status following interactions with the substrate and in relation to phase changes. To represent the association between these processes, a second Markov chain was introduced and connected to the above described cell cycle model (Fig. 1e). Basically, a *detached* cell is modelled as freely fluctuating into the medium and has a variable probability, depending on substratum and cell type features, of making a first stable contact with the plate thus becoming *attached*. Similarly, an attached cell has a given probability of starting a spreading process, thus becoming *spreading*, i.e. a state in which it reshapes by progressively flattening its volume and increasing its surface, until it becomes fully *spread*. Some phase changes in the cell cycle produce changes in attachment state, represented in Fig. 1e by dashed lines connecting, for example, *M* phase or apoptosis (*apo*) state with the *de-spreading* state. These attachment states can influence cell behaviour by acting on growth rates and motility and may be used as hints when graphically representing cells. In Fig. 1f, cell surface, plotted as a function of age for each cell of the previously described population, is shown to rapidly increase in the first few hours, as a result of cell spreading; after that, surface increases following volume increase and finally decreases, rapidly when cells undergo mitosis or more slowly when de-spreading during apoptosis.

**Fig. 2:**
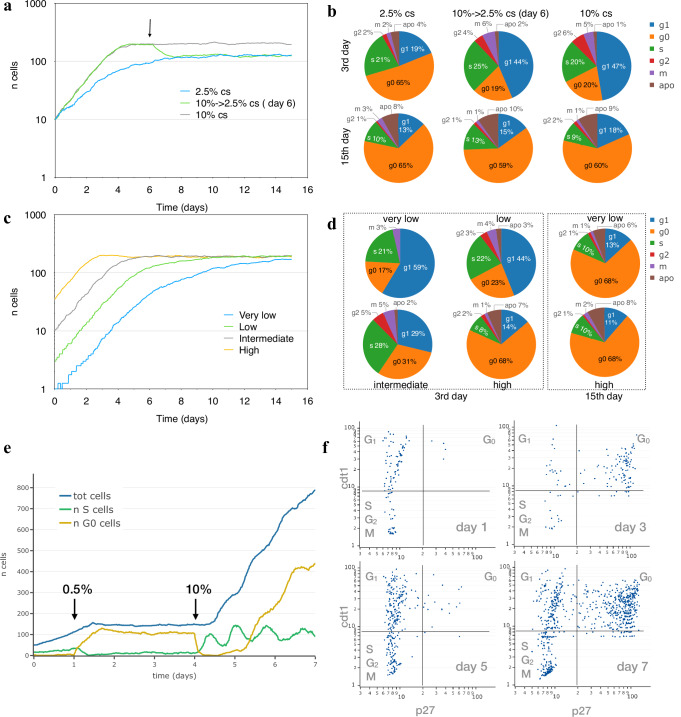
Analyses of cell populations simulated by the cell cycle progression model. **a** Growth curve, expressed as number of cells per 0.58 mm^2^, obtained for two simulated NIH3T3 populations followed for 15 days while growing at 10% (grey) and 2.5% (blue) calf serum, and for a third population (green) initially kept at 10 calf serum and moved to 2.5% on the sixth day (arrow). **b** Cell fractions in the different cycle phases on the 3rd day (exponential growth) and on the 15th day (stationary state). **c** Growth curve, expressed as number of cells per 0.58 mm^2^, for four simulated NIH3T3 populations seeded at four different densities (1, 4, 10, 50 per 0.58 mm^2^) and followed for 15 days while growing at 5% calf serum (grey). **d** Percentage of cells in the different cycle phases on the 3rd and 15th day. **e** Growth arrest of simulated population induced by serum starvation and rescue by serum re-addition. NIH3T3-like cells were simulated while growing under 10% FBS and, at day 1, starved by moving them to 0.5% FBS; on day 4 serum was restored to 10% and cells were followed for 3 more days. Total number of cells (blue line) and the number of cells in S (green line) and G_0_ (yellow line) phase are shown in the plot. **f** Snapshots of the simulated population in (**e**) at day 1, 3, 5 and 7 are reported by plotting each cell according to cdt1 and p27 concentration.

In Fig. 2a, two populations of NIH3T3-like simulated cells were followed for 15 days while growing at serum concentrations of either 10% or 2.5% calf serum together with a third one first growing at 10% and later at 2.5%, after a serum step down on the sixth day. The simulated populations show different growth rates for the two serum concentrations, and stop growing at different cell densities when the cultures arrive at confluence and cell numbers become stable; these patterns are very similar to those observed a long time ago in experimental cultures^53^; see Supplementary Fig. 1 for a graphic comparison. Consistently, the distribution of cells in the different cell cycle phases changes with time: on the 3rd day (exponential growth), about half cells are in G_1_ phase at 10% cs, but only 20% of cells are in G_0_; the G_0_ fraction is much higher (52%) in cells growing at 2.5% cs (Fig. 2b). This difference becomes much smaller when populations reach confluence and cell numbers stop increasing: under these conditions, cell cycle phase composition is very similar for the three analysed populations (15th day). These fractions are very close to those observed under similar conditions in experimental cell populations^54–57^; for cells exponentially growing at 10% serum, for example, Kues et al. report 67–73% cells in G0/G1 and 9–16% in S phase. In Fig. 2c, d simulated NIH3T3-like cells, seeded at four different densities, were “grown” for 15 days at 5% cs. During the earlier days, the observed percentage of G_0_ cells varies and progressively increases as cell numbers go up, but when, later in the experiment, the stationary phase is reached, most cells (~70%) are in G_0_ phase and cell numbers become independent of the initial seeding density (Fig. 2c, d).

Cells simulated in this way respond to different levels of serum by modifying their growth rate and progression through the cell cycle; their behaviour under more extreme conditions was tested to see whether they react by temporary or permanently arresting cell cycle progression. In Fig. 2e, NIH3T3-like cells were simulated initially as growing under 10% FBS and then as starved by reducing FBS to 0.5%; results show that while cells are kept at 10% FBS, they exponentially grow over time but, when starved by stepping down FBS to 0.5%, cells slow down until they stop increasing in number as a large fraction (79–85%) of cells enter G_0_ phase, a fraction very close to the 78–80% observed in experimental populations under similar conditions^47^. Cell rescue from starvation, by again raising FBS to 10%, causes cells to enter S phase and synchronously restart growth and proliferation until they reach confluence and slow down again. In order to monitor cell cycle modifications, the accumulation of two reporter proteins, cdt1 and p27, frequently used as markers for G_1_ and G_0_, respectively, was also simulated by relating their synthesis to intracellular state and propensity to phase changes. In Fig. 2f, where each simulated cell is positioned in the plot according to the concentration of the two proteins on the indicated day, for each of the different serum levels the results are consistent with curves in Fig. 2e and very similar to those reported in the literature for the same cell type under similar conditions^55,56^. Cells “grown” under 10% FBS (day1) mostly have low p27 levels, following a pattern typical of cycling cells, while cells kept in low serum mostly show high levels of both cdt1 and p27, a pattern typical of G_0_ phase (day3). Cells rescued by raising serum back to 10% gradually reenter cell cycle as confirmed by the reduced p27 levels (day5) which again go up on the 7th day when they start reaching confluence. Supplementary Figure 2 represents the same plots, using colour to highlight the phase of every single cell: in the vast majority of cases, the quantity of p27/cdt1 matches the cell cycle phase.

### Cell motion paths generated in silico accurately reproduce experimental cells moving on a culture surface

**Fig. 3:**
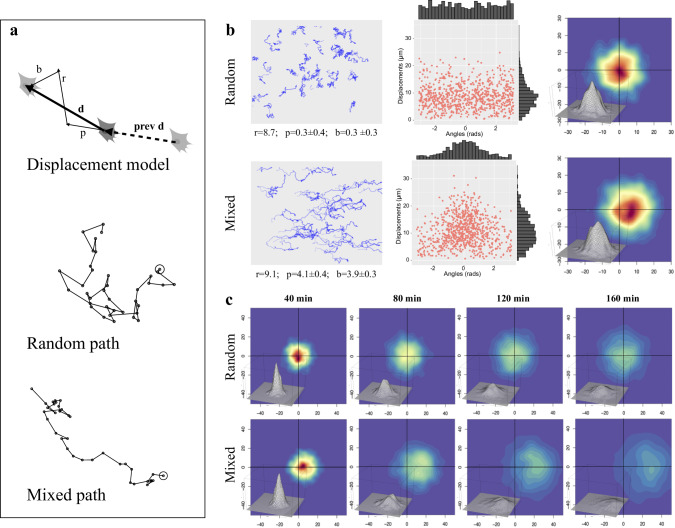
Simulation of cell migration and analysis of simulated cell paths. **a** Simulation of single cell displacement and full paths. The top schema shows the construction of a cell displacement (*d*), produced by combining a random vector (*r*) with a persistence one (*p*), characterised by a user defined module and the same direction as the previous cell displacement (*prev d*), and an external bias vector (*b*) along user defined direction. The lower two schemas report examples of a purely “Random” path and a “Mixed” one, in which directional persistence and bias have been added. **b** Paths and displacements produced by simulating two cell populations characterised by “Random” (top row) or “Mixed” (bottom row) movement. For each simulated population, the paths followed by each cell are reported in the left column, with, below each graph, the values of random, persistence and bias modules obtained by analysing them by using the three-component model^47^; an opacity gradient is used to mark the passing of time while drawing cell paths. In the middle column, displacement modules are reported as a function of direction, with their distributions reported as histograms along the two axes. The right column reports, for each cell population, cell displacements as two-dimensional kernel density distribution of shifts from previous positions using a colour code (warm to cold colours correspond to higher to lower density regions); a tridimensional representation of the same data is also included. **c** For each cells population, the same two-dimensional kernel density distribution is reported using shifts corresponding to four different time intervals, 40, 80, 120, 160 min.

Simulation of cell movement was setup by taking advantage of a previously published motion model, which describes movement of experimental cell populations as the combination of three vectors: a random, a persistence and a directional bias component^47^. The procedure, reported in Fig. 3a and in the *Methods* section, uses random, bias and/or persistence vectors to produce migration paths corresponding to completely diffusive, persistent or directionally biased movements, in various combinations. For each time interval, final cell displacement (*d*) is simulated by combining a random vector (*r*) with a persistence one (*p*), characterised by a user defined module and the same direction as the previous cell displacement (*prev d*), and a user defined external bias vector (*b*). While the combination of purely random displacement steps produces a relatively tortuous “Random path”, the “Mixed path” generated by more complex movement types is also characterised by directional persistence and/or bias along a given angle. The paths followed by two simulated populations, respectively characterised by “Random” and “Mixed” movement, are reported in the left column of Fig. 3b; in both cases, individual paths are different from each other and reproduce the variability typically observed in experimental cell cultures. Purely random paths, analysed according to the three-component model, show a 8.7 µm random module while persistence and bias ones are very close to zero; displacement lengths (centre column) show a long-tailed distribution peaking around the random module, with randomly distributed directions. Density distribution of displacement endpoints, plotted as starting from the origin (right column), shows the highest frequencies (indicated by warmer colours) around the origin and uniformly enlarges in all directions. Mixed movement, simulated by adding a persistence and a bias vector, produced more linear paths, directed towards the bias angle (0°), with estimated persistence and bias modules of about 4 µm. Accordingly, displacement directions are clustered around the bias angle while modules are higher than in random paths although still showing a long-tailed distribution. Cell displacements are strongly shifted in the direction of the bias vector but remain symmetrically distributed around the x axis. When simulated cell displacements are followed over time (Fig. 3c), their distribution progressively enlarge in all directions with time, maintaining the highest frequencies around the origin for random movements, and shifting it towards the bias direction for mixed population.

**Fig. 4:**
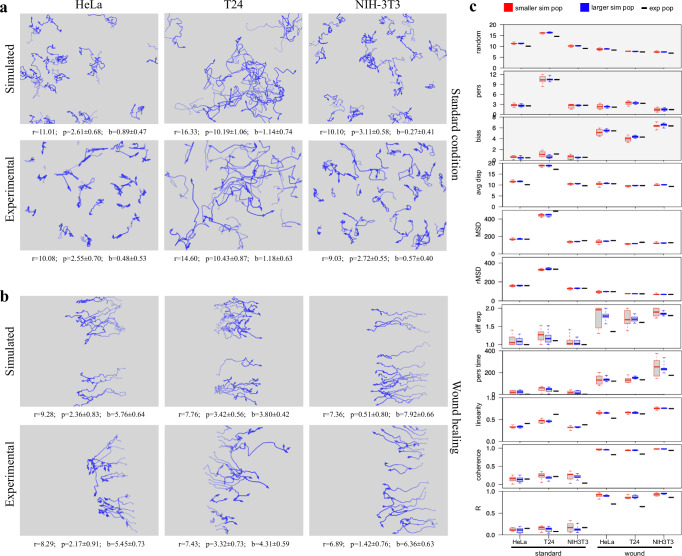
Simulations based on the movement model effectively reproduce cell paths observed in experimental cultures. For HeLa, T24 and NIH-3T3 populations, *x–y* coordinates registered during experimental time-lapse acquisitions and simulation experiments were used to draw paths followed by each cell, both under standard culture conditions (**a**) and during wound healing experiments (**b**); an opacity gradient is used to mark the passing of time while drawing cell paths. The calculated random (*r*), persistence (*p*) and bias (*b*) modules were reported under each graph. **c** For both standard and wound conditions, 10 populations were simulated starting either with the same number of cells as in the corresponding experimental one (red boxes) or with a standard number of 50 cells (blue boxes): for each movement parameter, a plot reports the distribution of its values among the simulated populations compared with the value obtained from the corresponding experimental one (black dashes). A grey background in the plot indicates parameters used to configure the features of the simulated cell populations.

In order to test the ability of the described procedure to reproduce the behavior of experimental cell populations observed in time-lapse experiments, the three-component model was used to calculate, from a panel of experimental cell cultures, random, persistence, and directional components to be used to generate movement in simulated populations. The panel includes a number of human tumour-derived cell lines (HeLa, T24, MDA-MB-231, PC3, A2058, A375, Calu and wm115) as well as two lines from murine embryonal fibroblasts, NIH-3T3 and NIH-Ras, the latter over-expressing a constitutively active variant of Ras, also known to be present in T24 cells. All lines were cultured and analysed while exponentially growing under standard conditions; for HeLa, T24 and NIH-3T3 lines, motion parameters were also determined while cells were moving in wound healing assay for 12 h after scratching the cell layer (Supplementary Table 2). Random, persistence and bias modules from experimental HeLa, T24 and NIH-3T3 populations were used to simulate motion behaviour of a small population of the same size as the experimental one, as well as a standard 50 cell population (Table 1). The results show very similar values for random, bias and persistence components for all populations of the same group and are well separated from those of the other groups (Supplementary Figure 3). Interestingly, simulated populations are very close to experimental ones also for additional movement parameters, not used to generate the simulated populations, such as average displacement module, final MSD and rMSD (random Mean Squared Displacement). In addition, simulations were also able to match the superdiffusive motion parameter *α*, consistently close to those obtained from the experimental populations and also higher when cells move responding to wound stimulus. In this case, persistence time, linearity and coherence as well as circular statistics R parameter also show correspondingly higher values. Paths generated by simulating standard populations also appear very similar to those originally observed in the experimental cultures (Fig. 4a). In addition, simulations reproduce many features of the experimental populations and maintain the differences between cell lines, with tortuous paths for HeLa, more linear and persistent ones for T24 and intermediate features for NIH-3T3 cells. Cells simulated while moving towards the wound produced directionally biased movements, similar both in appearance and as estimated parameters (Fig. 4b). Finally, in Fig. 4c, the variability of different simulated populations, generated from the same configuration parameters, was tested by producing, for each experimental population, ten simulated ones with the same number of starting cells and ten more with 50 starting cells each. For each of eleven parameters measured in both experimental and simulated populations, the distributions of values obtained from each group of simulated populations are displayed as box and whiskers plot alongside the corresponding values obtained from the experimental populations. The obtained distributions were rather tight for all tested parameters, even in populations characterised by lower cell numbers, and values from experimental cultures mostly fall within, or are very close to them. More variable values were only observed for diffusion coefficient and persistence time: also in this case the larger distributions observed in small populations become tighter when larger numbers of starting cells are used. It is worth noting that only three of the eleven parameters were used to configure the simulated populations (grey-background plots). Although not related to movement, replication time was also determined for the same populations with results reported in Supplementary Fig. 4: also in this case, the obtained distributions were rather tight and values from experimental cultures fall well within them.

**Table 1:** Similarity between simulated and experimental cell populations goes beyond the parameters used to configure the simulations

Experimental and simulated HeLa, T24 and NIH-3T3 populations under standard culture condition and in wound healing experiments were analysed and different parameters were calculated, including random module, bias and persistence modules normalised against the corresponding random module, average displacement, MSD and random MSD (rMSD) per 40 min, diffusion exponent (α), persistence time (p), linearity, coherence and R parameter. In the table, cells with a grey background contain parameter values obtained from the experimental population and used to configure simulations.

### From reproducing cell subpopulations to predicting conditional cell behaviour on a culture plate

**Fig. 5:**
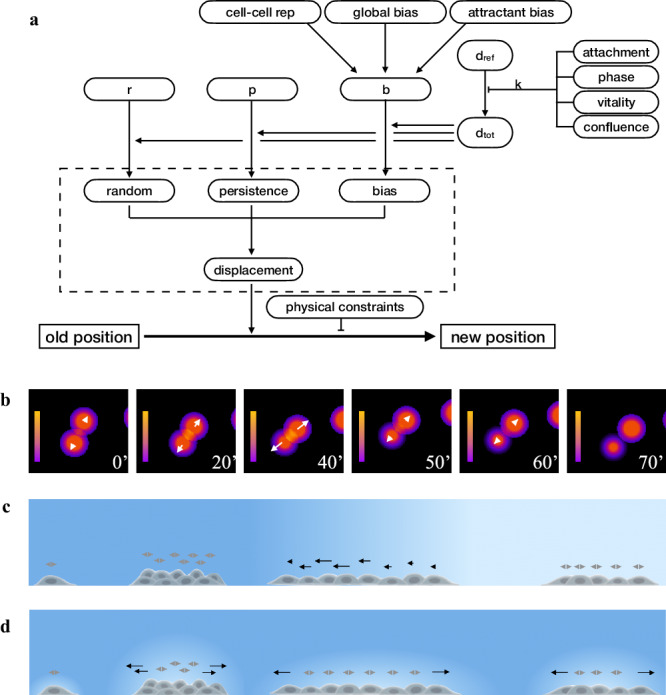
Schematic representation of the movement model. **a** The extended motion model used in the simulator builds on the original three component model, contained in the dashed box, to simulate the influence of internal and external factors on cell movement. *d*_*tot*_ is the maximum displacement for a defined time interval and is derived from *d*_*ref*_, assumed to be the maximum potential displacement obtained for the same cell type under an “optimal” set of conditions; *r*, *p* and *b* indicate how much of *d*_*tot*_ is necessary to produce the corresponding random, persistent and bias components. By modifying these parameters, a wide range of factors can influence cell displacement and its final position; examples are cell cycle phase, attachment state, level of vitality and of local confluence, the presence of physical constraints and “local” bias, like repulsion from neighbouring cell or attraction due to the presence of an attractant gradient in the plate. **b** Cell-cell repulsion gradient represented as colour shades corresponding to cell influence on the surrounding environment. For each cell, the repulsion vector is reported as a white arrow along the straight line between two neighbouring cells and whose module depends on gradient. The time interval between the first three images was 20’ intervals, while the others were taken at 10’ interval. **c** Effect of a relatively sharp attractant gradient on cell movement. For each cell, the bias vector is reported as an arrow whose module depends on the difference in attractant level over a distance corresponding to the size of the cell body. **d** Effect of dynamic self-generated local gradients on cell movement. The bias vector is reported as an arrow with a module which, as in **c**, depends on attractants level, which, in this case, is locally modified by the presence of cells able to degrade the attractant molecule in its immediately vicinity: higher cell concentration produces greater gradient than sparse cells. In both panels, a more intense blue indicates higher attractant level.

Unlike the previous simulations, where populations or subpopulations are homogenous and cell-to-cell differences mostly derive from apparently random individual variability, the behaviour of different cells growing on a culture dish is more likely not homogenous and changes according to intracellular state and/or local external conditions, thus giving rise to a potentially large number of different subpopulations within the same culture. To reproduce this cell-by-cell variability, the above-described motion model was extended to include the ability to modify speed, directionality, and other features according to the presence of an attractant, repulsion from neighboring cells, attachment state, and cell cycle phase. The method for generating movement was therefore modified to take into account the many factors able to modify movement, while calculating the displacement of each individual cell (Fig. 5a). Basically, random (*rand*), persistence (*pers*) and bias (*bias*) modules, obtained from an experimental population, were respectively assumed to be the result of the following expressions:

2 $${rand}=r\cdot{d}_{{tot}}$$

3 $${pers}=p\cdot{d}_{{tot}}$$

4 $${bias}=b\cdot{d}_{{tot}}$$

where *r*, *p*, *b*, whose sum is equal to one, correspond to the fraction respectively used by random, persistence and bias components of *d*_*tot*_, i.e. the “maximum potential displacement” of a given cell type, travelling under a given condition. The average *d*_*tot*_ value observed in the experimental population is in turn assumed to derive from a reference value, *d*_*ref*_, through a coefficient *k*, representing the cumulative effect of cell confluence, limited nutrients or growth factor availability, or other inhibitors of cell movement. When generating movement with the extended motion model, for each simulated cell, *d*_*tot*_ is re-calculated by multiplying *d*_*ref*_ for a *k* value depending on various factors including cell cycle phase, attachment state, level of vitality and local confluence. Similarly, *b* is modified by taking into account the effect of factors able to influence the final bias vector, such as global bias, repulsion from neighbouring cells or bias due to the presence of an attractant gradient. Re-calculated *r*, *p*, *b* and *d*_*tot*_ are now used to obtain the new random, persistence and bias modules, which, combined according to the three component model, give the final displacement. Finally, cell position is modified using the obtained displacement and optionally taking into account the presence on the culture surface of physical borders or other constraints which slow down or completely block cell movement.

As with the extended motion model it is relatively easy to compose the effects of different factors simultaneously affecting the movement of every single cell, support was added for short range cell–cell repulsion and locally variable gradients of attractant molecules.

Cell-cell repulsion was determined by identifying, for a given cell, the neighbouring cells within a given distance and by calculating, for each of them, a repulsion vector along the straight line between the two cells with a module depending on distance, cell speed and sensibility to repulsion by other cells (Fig. 5b).

Similarly, to simulate the effect of gradients of attractant molecules, another bias vector is determined from the difference in attractant level between front and back of a given cell. This vector works very nicely for short and/or sharp gradients able to produce concentration differences at distances comparable to the size of a cell body (Fig. 5c). Support for shallow gradients was introduced by considering the effect of dynamic self-generated local gradients as those previously described in literature^58,59^, which assume that a shallow or even completely flat gradient is locally reinforced by the ability of cells to degrade the attractant molecule in their immediately vicinity (Fig. 5d). Such local gradients have been shown by different authors to be more robust, to work across greater distances and to be better suited to represent the effect of the local environment than purely passive cell responses^60–66^.

**Fig. 6:**
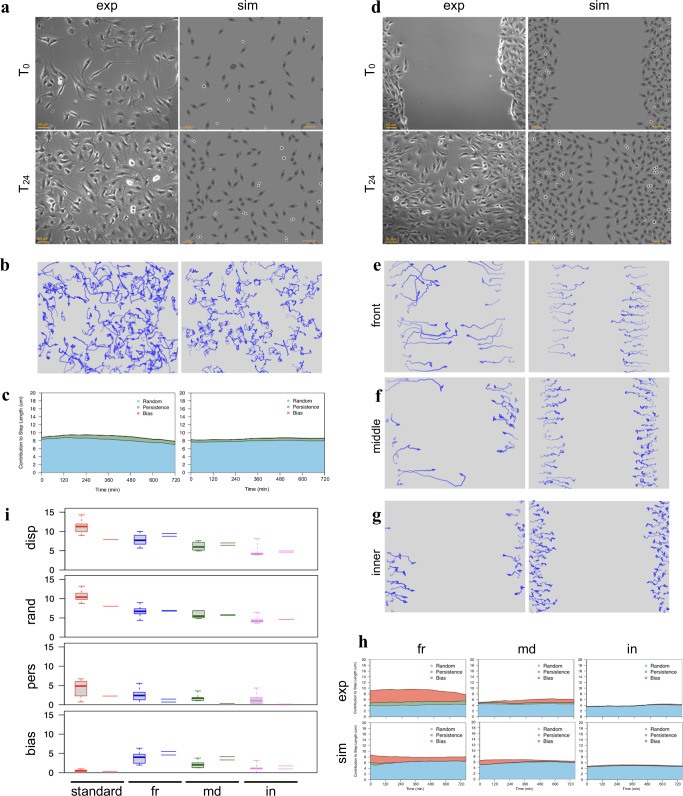
Simulation of NIH3T3-like cells moving under wound healing experiments. Movement parameters obtained from the experimental cell population, kept under standard conditions, of panels (**a-c**) were used to produce synthetic populations moving in a wound healing experiment in panels (**d–h**). **a** Phase contrast images taken at 24 hour distance of cells exponentially growing under standard culture conditions and in absence of any directional bias (left side images), compared to a simulated cell culture “grown” under the same conditions and rendered at the same times using a phase-contrast inspired symbolic representation (right side images). **b** Movement paths of experimental and simulated cells during the 24 h period, drawn by using an opacity gradient to mark the passing of time. **c** Contributions to cell displacement of random (blue), persistence (green) and bias (red) evaluated for experimental and simulated cells by analysing, at each time point, overlapping 12-h windows. **d** A wound healing simulation, where the cell population uses parameters from the experimental population of panel (**a**), is compared to a wound healing experiment featuring an independent experimental population. For both simulated and experimental populations the images are organised as in panel (**a**). **e–g** Movement paths for experimental and simulated sub-populations corresponding to cells located after the wound at the front (**e**), middle (**f**) or back (**g**), drawn, as in (**b**), by using an opacity gradient to mark the passing of time. **h** Average contributions to cell displacement of random (blue), persistence (green) and bias (red) evaluated over time, by separately analysing overlapping 12-h windows for experimental and simulated cells located at the front (fr), middle (md) and back (in). **i** Distribution of displacement, random, persistence and bias values obtained for experimental cells from 5 populations under standard culture conditions (red boxes), 16 populations at the wound front (blue boxes) and 6 intermediate (green boxes) and internal populations (violet boxes). The corresponding values from the simulated populations are reported as dashes.

The extended motion model can effectively generate, as shown in Fig. 6, the variety of different cell subpopulations typical of wound healing experiments, starting from the parameters determined from an experimental NIH-3T3 culture exponentially growing under standard conditions and moving in absence of any directional bias. As shown in Fig. 6a, the original experimental culture (left panels) was analysed to obtain the “base parameters” *r*, *p*, *b* and *d*_*ref*_, which were then used to produce a similarly numbered population of simulated cells (right panels) which, “growing” under the same conditions, reach a comparable number of cells in 24 h and, at the same time, move along similar paths (Fig. 6b). The contributions to displacement of the three components, evaluated over time (see *Methods*), show a pattern similar to the experimental population, with an average displacement mostly made of a random component, with a smaller persistence and no bias (Fig. 6c). In Fig. 6d, the same “base parameters” were used to simulate a wound healing experiment where cells are grown at high density, to make them initially produce an highly confluent “layer” which is then subjected to a scratch. While “growing” at high density, *d*_*tot*_ values are reduced by lower nutrient availability or specific cellular states: for example, cells move slower when in G_0_ or during apoptosis. After the scratch, cells by themselves tend to move towards the free space by diffusion reinforced by cell-cell repulsion. The “attraction” by the wound was simulated by adding two further components, both simulated using dynamic self-generated local gradients, which reflect the effect of free space and the attraction by cell debris produced in the wound area soon after the scratch. The first uses a flat long-lived attractant uniformly present in the plate to represent the effect of different serum factors; the second, with a relatively short half-life, simulates the effects of molecules released by scratch damaged cells^67–73^. The two gradients produce two bias vectors which, combined with others, such as that introduced by cell to cell repulsion, result in a final bias, different for each cell. The resulting behaviour is much more complex than in the starting population and leads to wound closure (Fig. 6d, right panels) with timing and patterns very similar to those of an experimental NIH-3T3 population observed under the same conditions (Fig. 6d, left panels). Simulated cell paths show a progressive decrease in length and linearity as the distance from wound front increases (Fig. 6e–g) and front (*fr*), middle (*md*) and inner (*in*) subpopulations, separately analysed produce results which closely resemble those from experimental cultures. The contributions to displacement of the three components, evaluated over time, also show patterns close to those of experimental populations, with longer displacements for front populations mostly due to higher bias, much lower displacement and bias values for internal cells with intermediate values for middle cells (Fig. 6h). When movement parameters obtained from the analysis of the simulated populations are compared to a larger number of experimental populations in the same conditions, total displacement as well as random, persistence and bias components are within or very close to the range of experimental values (Fig. 6i).

### Setting up and executing simulations within *SimulCell* web application

**Fig. 7:**
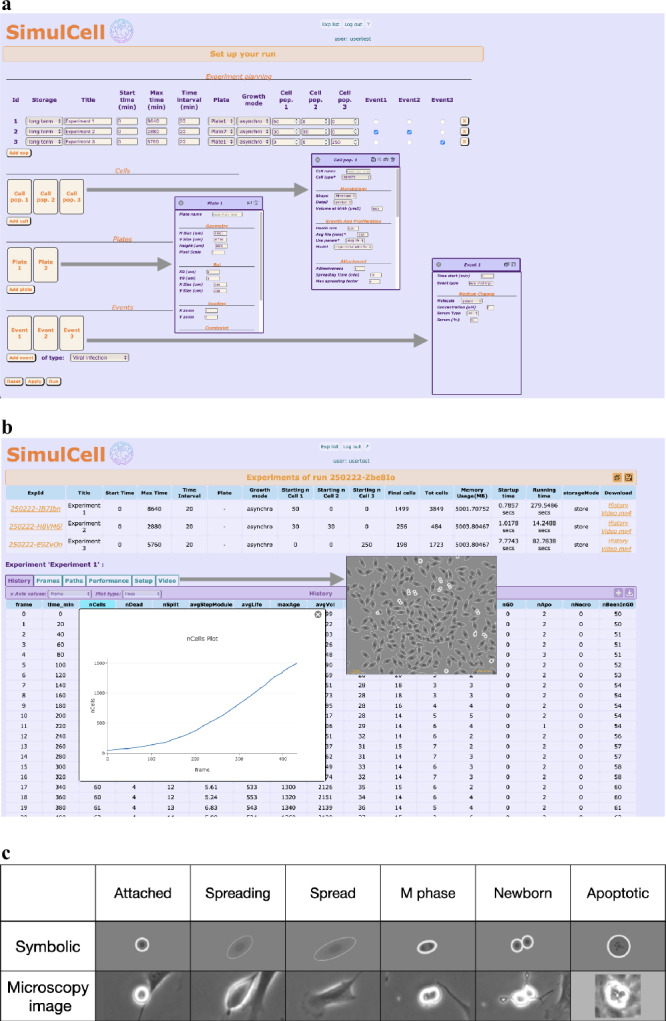
*SimulCell* web application. **a** The setup page is used to define a multi-experiment run using the available plates, cells and events and gives access to all parameters needed to run a simulation through dialog sections customised for plate, cell and event. **b** The result page gives access to the simulation results using customised tables and plots and showing videos to display cell behaviour. **c** Simulated “symbolic” representations of cell morphology compared to phase contrast images of experimental NIH3T3 cells under similar conditions: cells are visualised as oval shapes, whose size and elongation depend on cell features as well as simulated volume and degree of spreading; as for their experimental counterpart, border colour is dimmer in fully spread cells and becomes thicker and clearer when cells de-spread during nuclear duplication and division or apoptosis.

The described models and procedures can be used together to produce simulated populations within *SimulCell*, a web application designed to setup and run simulation experiments and to quickly analyse the results. Input data can be manipulated using standard dialog boxes, organised in sections containing experiment, plate, cell and event parameters (Fig. 7a). During the calculation, the application provides support for monitoring the process, by showing images of the simulated cultures and plots reporting number of cells or other data. After the calculation, the result page displays major parameters and statistics organised in different tables: *History*, containing summary results for each time point, *Path* and *Frame*, where data are respectively organised by cell and time interval, in addition to *Setup* and *Performance*, containing the major simulation parameters used to setup the experiment and run duration and other performance indicators. Still within *SimulCell*, many different plots can be generated to visualise data as custom graphs designed to highlight specific data features (Fig. 7b) and images and videos are used to display cell behaviour and morphology during the simulated experiment. Within *SimulCell*, as also in images shown in Fig. 6, cell visualisation uses a “symbolic” representation, where cells are simple geometric shapes, which change depending on cell type, status and condition. Cell body is represented by a circle or an ellipse whose size conveys information about volume, degree of spreading and cell polarisation. Images use a grey level colour set, reminiscent of phase contrast microscopy, to let the user recognise cell growth, attachment status, nuclear duplication and division in a familiar way; some examples are reported in Fig. 7c.

**Fig. 8:**
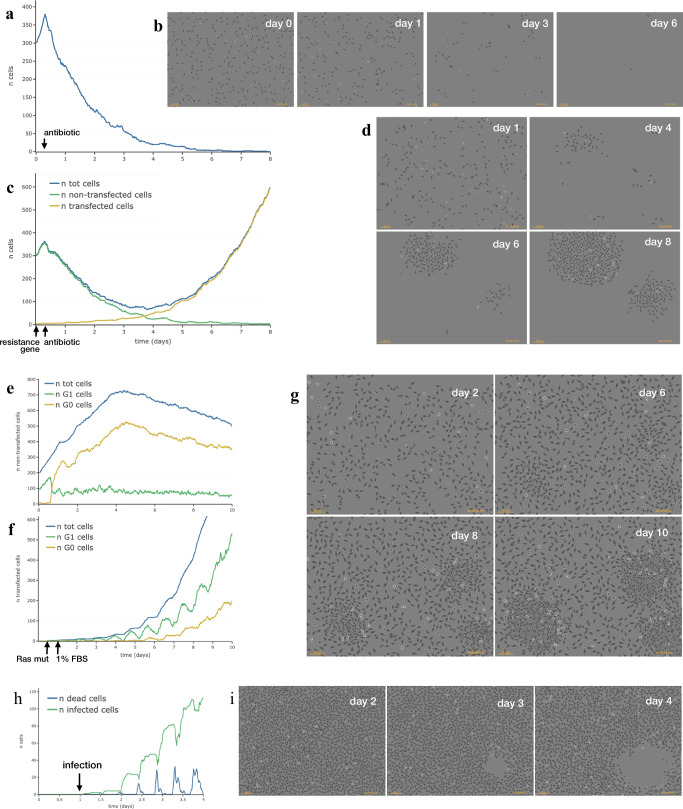
Experiment examples performed within *SimulCell.* **a** Number of simulated NIH3T3-like cells while responding to an antibiotic added to the medium at time 6 h and followed for 8 days. **b** Phase-contrast like images of the same population on days 0, 1, 3 and 6. **c** Production of antibiotic-resistant clones by DNA transfection. After transfection with an antibiotic resistance gene at time 0, the number of total simulated NIH3T3-like cells (blue line), non-transfected (green line) and transfected cells (yellow line) is reported over time while cells respond to an antibiotic added to the medium at time 6 h. **d** Phase contrast like images of the same population on days 1, 4, 6 and 8. **e–g** Production of foci by overgrowth after transformation by exogenous DNA. The total number of cells (blue line) and the number of cells in S (green line) and G_0_ (yellow line) phase after transfection with constitutively activated Ras gene at time 12 h and serum step down to 1% FBS ate time 24 h is reported for non transfected (**e**) and transfected cells (**f**). **g** Phase contrast like images of the same population on days 2, 6, 8 and 10. **h** Number of dead (blue line) and infected (green line) simulated VeroE6 cells after infection with a lytic virus (9 h replication time). **i** Phase contrast like images of the same population on days 2, 3 and 4.

Figure 8 shows some simulations executed within *SimulCell*, which highlight how cell behaviour is affected by the specific conditions defined while setting up the experiment and each individual cell agent integrates different aspects of cell behaviour and reacts to local environment changes produced by other cells or resulting from external physical or chemical events.

In the example reported in Fig. 8a, b, the vitality of each cell is differently affected by an antibiotic drug added to the medium and simulated cells respond by progressively dying until they completely disappear over time. When the same cells are “transfected” with an antibiotic resistance gene, some acquire the new gene (Fig. 8c, yellow line), become resistant to the antibiotic and grow and proliferate in its presence, producing resistant clones (Fig. 8c, d) (Supplementary Movie1).

In Fig. 8e–g, simulated NIH3T3-like cells were modified by acquiring a constitutively active Ras gene by transfection and later moved to 1% FBS (Supplementary Movie2). Under these conditions, cells keep growing although at a lower rate because of reduced serum stimulation and, after a few days, some cells, having acquired the mutated Ras gene, accelerate growth and produce clonal foci which after a while start overcoming the surrounding cells (Fig. 8g and Supplementary Fig. 5). The plots show that in low serum non-transfected cells slow down their growth and enter G_0_ phase, unlike the few transformed cells which actively proliferate and in a few days start a fast exponential growth and become the predominant cell population concomitantly with the appearance of foci in the culture.

Finally Fig. 8h, i, shows the effect of infecting simulated cells with a lytic virus (Supplementary Movie3). Here, cell agents were configured with parameters from VeroE6 cells and virus infection was assumed to lead to cell lysis in about 9 h and to release new infective virus particles in the surrounding area. The results show that 24 h after infection (day 2) the layer is still confluent, but that starting from day 3, a lysis plaque becomes visible and enlarges over time following the few replication cycles corresponding to the sequential waves of infection caused by subsequent local releases of new virus particles.

## Discussion

Modelling the enormously variable behaviour of a population of eukaryotic cells is an inherently difficult task, given the inevitable differences between single individual cells. In many cases, the simple observation of cell populations of different sizes allows to define a “collective” behaviour, usually relatively easy to analyse and even predict once a number of environmental parameters have been established. But in a real cell population, the “collective” behaviour derives from the combination of many independent individual cells, each of them behaving according to its own microenvironment and following basic rules defined by physics and chemistry: an approach completely based on this principle could, if at all conceivable, completely define all cell features and activities. Although such a detailed approach appears to be well beyond our current abilities, different aspects of cell behaviour, such as cell proliferation and movement, have been the target, in recent years, of a search for parameters and models able to describe them in good detail from the phenomenological point of view^2,5,74,75^. Such models have been used as a base to generate, by in silico simulation, new populations which, to a given extent, can resemble experimental ones and may be challenged for testing hypotheses^6,26,30,37^. Observing the behaviour of such simulated cultures may turn out to be particularly useful when the experimental system is complex, components and parameters increase in number and the outcome is consequently more difficult to predict analytically.

The agent-based simulation system proposed in this work goes along these lines, simulating single cells which, interacting with each other and with their environment, individually contribute to generate a simulated cell population which can accurately reproduce movement and many other features observed in experimental cell cultures. Movement simulation builds on a previously developed motion model^47^, which was here further extended to add the necessary flexibility: within the system, the cells, defined by “base” parameters obtained from the analysis of a given population, react to environmental changes, by modifying the “base” parameters taking into account their own intracellular state and local external conditions. This approach gives rise to a potentially large number of different cells and/or subpopulations within the same culture. Cells simulated in this way, can, all at the same time, feel cell-to-cell repulsion, choose their direction reacting to the presence of attractant molecules and modify movement according to cell metabolism, attachment state and cell cycle phase; while doing that, the same cells control growth, survival and replication, as well as cell cycle transitions, also in relation to local cell confluence and presence of nutrients or drugs in the medium. In this way, cell-to-cell differences derive from random individual variability but are also due to a non-random differential behaviour, which reflects intracellular state and/or local external conditions and can give rise to different subpopulations within the same culture.

Simulation planning and execution takes advantage of a web application, *SimulCell* (http://simulcell.ceinge.unina.it/), which provides a standard setup, but where most parameters may be modified and a large number of cell functions and features can be easily graduated or simply turned on/off. *SimulCell* was designed to be an environment where models for movement, growth and proliferation are already available, and can be used to test the behaviour of cultured cells by users who can access parameters for different cell types and easily modify them. It provides a small collection of basic parameters, corresponding to a set of experimental cell types and/or populations, and can be used to modify most parameters defining cell behaviour, culture plate specifications, medium components, geometry of the acquisition area. Events occurring during the simulation are also set up through the interface, including changes in serum type and level, addition or removal of physical borders, mechanical injuries, addition of attractant molecules or drugs; in the same way, even simple acquisitions of exogenous genes or a basic “viral” infection procedure can be used in experiment planning. Being available as a freely accessible web application, *SimulCell* requires no installation or package dependence and can run, maintaining performance, independently from the available computer hardware or operating systems. These features possibly contribute to make cell simulation more easily accessible by users with no programming experience than other currently available simulation tools. In this sense *SimulCell*, unlike other frameworks for building models such as *CompuCell3D* and *PhysiCell*, does not require users to write their own code or build and manage more or less complex XML models, although a large number of parameters may still be modified and different combinations may be easily tested, stored and replayed; in this sense, the system may turn out to be usable when testing hypotheses or trying to answer questions of the “What if…?” type. *SimulCell* is also a potentially useful demonstration tool for academic courses, where it should be relatively easy to set up typical experimental situations involving standard cell cultures to test cell response in different conditions and to try experimental setups difficult or impossible to reproduce in a wet-lab practical lesson. The use of *SimulCell* within an educational context could be particularly indicated as, being a web tool, its performance is relatively independent from the browser or the computer hardware available to the user: of course, when long tables or large movies are produced, better hardware and a faster browser can result in a more pleasant experience.

Although the system is effective at simulating motion and proliferative behaviour, its use is necessarily restricted by the planned aspects of cell behaviour. For example, the 2D nature of the current model does not account for complex 3D interactions between cells and with the extracellular matrix, a feature which would obviously help while working with tissue-like structures. In addition, in its present form the system only keeps track of a few protein molecules: more molecular simulations would certainly help create more control points for internal signalling or external factors. However, the modular architecture makes it relatively easy to extend, thus opening up future perspectives. A welcome addition, for example, would be a better definition of cell morphology, which could be of help in both simulation and result visualisation phases. Similarly, interfacing with an external molecular simulation tool would add support for a larger number of simulated molecules, a useful feature when investigating physiological or pathological situations involving regulatory molecules and signalling pathways^76–79^.

## Methods

### Cell culture

Cells were propagated in Dulbecco’s Modified Eagle’s Medium (DMEM) supplemented with L-Glutamine (2 mM), 10% foetal bovine serum (FBS), penicillin (10 U/ml) and streptomycin (10 ng/ml) and maintained at 37 °C and 5% CO_2_. Cells were transferred by detaching them in trypsin/EDTA (trypsin 0.05% and 0.53 mM EDTA) and collecting them with a complete culture medium. After centrifugation at 1200 rpm for 5 min, pellets were suspended in fresh medium, properly diluted, and plated again.

The cell lines used for time-lapse acquisitions include murine fibroblasts NIH-3T3^80^ and NIH-Ras produced by transfecting RasV12 into NIH-3T3^81^, and human immortalised cell lines HeLa from cervical cancer^82^, T24 from bladder carcinoma^83^ MDA-MB-231 from breast cancer^84^, A2058, A375 and wm115 cells from melanoma^85–87^ and PC3 and Calu cells from prostatic and lung adenocarcinoma, respectively^88,89^.

To investigate random movement ability, 25000 cells/well were seeded in 12 well plates and maintained in complete medium at 37°C and 5% CO_2_. After 16–18 h, the plate was placed in the incubator chamber of the microscope. For wound healing assays, cells were seeded in confluent monolayers by plating 250000 cell/well in 12 well plates in complete medium; 24 h after plating the cell layer was scratched with a sterile pipette tip.

### Data acquisition

Phase contrast images (objective 10x) of different samples have been acquired at 10 min interval for 24 h or more by using a Zeiss Cell Observer system, composed by an inverted microscope (Axiovert 200 M), an incubator chamber that maintains the temperature at 37°C and CO_2_ partial pressure of 5%, and a digital camera (Axiocam H/R or Hamamatsu Orca Flash). A motorised stage was used to permit prolonged automatic acquisitions at different positions. For this work, digital frames were typically acquired as 16 bit images of 650×514 pixels. The pixel scale of the acquired images is 0.767 pixel/µm, obtained by acquiring with the same system an image of a Burker chamber with known measures.

Cell displacement was tracked by using a semi-automated procedure available within *MotoCell*^90^. The tracking procedure allows to collect cell positions (in terms of x/y coordinates) at different times (frames) to construct for each cell a path characterised by an origin (start, newborn, found, gone in) and a destiny (split, dead, lost, gone out). The registered data are stored in a text file that can be read by *MotoCell* to perform the quantitative analysis.

### Mathematical and statistical analyses

Mathematical and statistical analyses were carried out within the R environment. The function *kde2d* (*MASS* package) has been used to compute the two-dimensional density distribution of cell positions (x and y coordinates), graphically represented by using the *image* and *persp* functions (*graphics* package), for 2D and 3D representations, respectively. In addition*, stats* package from the basic configuration as well as the external package *ggplot2* have been used to produce most graphics. Curve fitting has been done by using *nls* (non-linear least squared) function.

### Determination of movement parameters

The random, persistence and bias components of analysed cell movements were evaluated using the model and procedure described in Toscano et al. (2022)^47^. Diffusive behaviour was evaluated by collecting mean squared displacements (MSD) corresponding to progressively increased time intervals and by fitting the function ${MSD}=k\cdot{t}^{\alpha }$ to the data, using weights proportional to the number of averaged squared displacements. Persistence analysis was carried out by fitting the model initially proposed by Fürth et al. in 1920^91^and described by Alt et al. in 1990^92^, where the relation between *MSD* and time (*t*) is given by the following equation:

5 $${MSD}=2{S}^{2} \cdot{P}\cdot\left[t-{P}\cdot\left(1-{e}^{\frac{-t}{P}}\right)\right]$$

where *S* is the root mean squared speed and *P* is the directional persistence time, i.e. the time in which cell movement tends to persist in the same direction.

Linearity was calculated as the ratio of net displacement (i.e. the distance between the starting and end point) to path length. For a given population, linearity is the average of linearity values independently calculated for each cell. The coherence parameter of a population was measured as the ratio between the length of the resulting vector, obtained by composing the displacement vectors for each cell path and the sum of the single net displacement vector lengths. Circular statistics analysis was used to evaluate the distribution of cell displacement directions. The displacement vectors were combined without taking into account vector modules to obtain a resulting vector having its origin in the centre of a circle with unit radius. Its direction is defined as the average angle, whereas its length is named linear dispersion coefficient (R), which is descriptive of the dispersion of the angles around the average. R value ranges between 0 and 1: values close to 0 indicate a uniform angle distribution with no directional bias; larger values are obtained when displacement angles are clustered around a given direction, reaching 1 in the special case of all identical angles.

The average scalar contribution of random, persistence and bias components to displacement length were calculated by averaging, for a given time window, the projections of each of the corresponding vectors onto the displacement direction.

### Calculation of cell cycle state transitions

Cell cycle state progression was simulated by using Markov chains, where each cell moves from one state to the next by using probabilities calculated by combining the effects of number of factors able to influence the transition. At each time point, the probability (*prob*) of moving to a different status in a given time (*probTime*) is adapted to the simulation time interval (*timeInt*) according to the following formula:

6 $${pro}{b}_{{timeInt}}=1-\left({(1-{prob})}^{\frac{{timeInt}}{{probTime}}}\right)$$

The effect (*eff*) of each enhancing or limiting factor affecting the transition is reported within the 0–1 interval by using the logistic function:

7 $${eff}=\frac{1}{1+{e}^{k({X}_{c}-{X}_{{ref}})}}$$

where *X*_*c*_ in the current value of the parameter which influences the transition, *X*_*ref*_ is the mean of logistic function and is the value at which there is a 50% probability to go the next state and *k* is the logistic growth rate or steepness of the curve.

### Generation of cell displacement from movement parameters

Cell movement is simulated by using three parameters corresponding to the length of random, persistence and bias components: during each time interval, a ten step random walk is calculated to generate a vector of variable direction and module which is optionally combined with a persistence and a bias vector. The three components may be provided by the user, but more often are the result of the evaluation of experimental paths according to the method and procedure described in Toscano et al. (2022)^47^. In alternative, the two-level approach described in the Results section was used to obtain movement parameters starting from the “base parameters” (*r*, *p*, *b* and *d*_*tot*_). This last method allows to adapt experimentally determined parameters to culture conditions other than those used to acquire them and, at the same time, is used to modify them for each individual cell, taking into account local medium changes or other factors affecting cell movement. In addition, the global bias represented by *b* value, is combined with additional bias vectors due to repulsion from neighbouring cells and/or the presence of an attractant gradient. Modified *b* value, together with *r* and *p* values, are then rescaled to bring their sum back to 1 and used to obtain the new random, persistence and bias components to be used to calculate the final displacement. Support for short range cell-cell repulsion is introduced as an additional bias: for each cell, the neighbouring cells within a given radius are identified and, for each of them, a repulsion vector is calculated along the straight line between the two cells and with a module depending on distance, cell speed and sensibility to repulsion by other cells. Another additional bias is used to support local attractant gradients determined as difference in attractant level between cell front and back. These levels are optionally adjusted to produce a dynamic self-generated local gradient to simulate cells able to degrade attractants.

### *SimulCell* development

*SimulCell* has been built by using the PHP programming language and using an object oriented approach which allowed the development of a strongly modular program. A schematic representation of the architecture of the simulation system in terms of objects and data flow is reported in Supplementary Figure 6. The execution of a simulation run is started by an *expRunner* object using parameters obtained from one or more *expSetup* objects, originally setup by the user through the *SimulCell* graphical interface or other means. For each *expSetup*, the *expRunner* creates one *experiment* object, responsible for time management and for processing simulation data, recorded at each time interval using a *movie* object. An *experiment* creates a *plate* object, which works as a “dynamic” container of cells and represents the ever changing environment in which they live, move and proliferate. A *plate* has properties like geometry, surface and medium, whose components are updated at each step and can influence cell behaviour. According to the input parameters defined by the user, different *cell* objects are added to the *plate*, each of them set up starting from a parameter library derived from the analysis of experimental cell populations (some are reported in Supplementary Table 2) and modified according to user choices contained in the *expSetup*. The *cell* object contains the current state of the cell and provides most functionality in terms of functions, responsible for implementing the models defining cell behaviour. Cells use a plate-owned *cellGrid* to manage and calculate cell-cell and cell-device distances and interactions. A general *event* class is used to contain the basic features of any event and is further extended to produce specific events: *update, mediumChange, moleculeAddition, gradientAddition, contraintRemoval, scratchOnPlate, transfection, viralInfection*, are examples of currently available events.

In its present form, *SimulCell* runs as a web application in an apache2-PHP environment and benefits to some extent from multi core processing. Examples of execution times obtained from a 12-core M1 processor are reported in Supplementary Table 3. Simulation of a 100 cell starting population for 5 days produces a final population of over 1600 cells in less than 3 min. Execution time per cell increases linearly with simulation time; similarly, it linearly increases with the number of starting cells, with an average of 0.04-0.06 seconds per cell per day, corresponding to over 1400-1900 cell updates per second of simulation.

### Representation of cell morphology

Within *SimulCell*, the generated simulations produce, in addition to text based outputs which report the results as tables or plots, a dynamic graphical representation of cells that can be used to build image stacks and mp4 movies. Here cells are represented by using a “symbolic” representation where cells use simple geometric shapes which change depending on cell type, status and condition. Cell body is represented by either a circle whose radius depends on cell volume and degree of spreading or an ellipse which, in addition, has a variable level of elongation to indicate cell polarisation along one axis. Images use grey level colour sets, reminiscent of phase contrast microscopy, where changes in shape, border colour, cell opacity and contents are used as visual hints to help the user better recognise cell growth, attachment status, nuclear duplication and division.

## Supplementary information

Supplementary Information

Movie1.

Movie2.

Movie3.

## Data Availability

Procedures and data used to generate the described experiments are provided in the main text, in figures and Supplementary Information; access to simulations based on the described models is freely available through the *SimulCell* web page (http://simulcell.ceinge.unina.it/), together with a library of sample cell types. Any additional information may be obtained from the corresponding author upon request.

## Code availability

The simulations described in the manuscript were produced through *SimulCell* (http://simulcell.ceinge.unina.it/), a web application used during the project to test the described algorithms and procedures and to produce the results of the example runs. Algorithms and procedures are described in text and figures provided in the Manuscript and in the Supplementary Information files. The authors will respond to all reasonable requests from the readers after publication.

## References

[1] Kramer, N. et al. In vitro cell migration and invasion assays. *Mutat. Res.* **752**, 10–24 (2013). 22940039 10.1016/j.mrrev.2012.08.001

[2] Masuzzo, P., Van Troys, M., Ampe, C. & Martens, L. Taking aim at moving targets in computational cell migration. *Trends Cell Biol.* **26**, 88–110 (2016). 26481052 10.1016/j.tcb.2015.09.003

[3] Ren, J. et al. Recent advances in microfluidics-based cell migration research. *Lab Chip* **22**, 3361–3376 (2022). 35993877 10.1039/d2lc00397j

[4] Hu, Y., Becker, M. L. & Willits, R. K. Quantification of cell migration: metrics selection to model application. *Front. Cell Dev. Biol.* **11**, 1155882 (2023). 37255596 10.3389/fcell.2023.1155882 PMC10225508

[5] Toscano, E. et al. Methods and computational tools to study eukaryotic cell migration in vitro. *Front. Cell Dev. Biol.* **12**, 1385991 (2024). 38887515 10.3389/fcell.2024.1385991 PMC11180820

[6] Barh, D. et al. In silico disease model: from simple networks to complex diseases. *Animal Biotechnol.* **441**10.1016/B978-0-12-811710-1.00020-3 (2020).

[7] Johnson, G. T. et al. Building the next generation of virtual cells to understand cellular biology. *Biophys. J.* **122**, 3560–3569 (2023). 37050874 10.1016/j.bpj.2023.04.006 PMC10541477

[8] Carvajal-Rodríguez, A. 1LocusSim a mobile-friendly simulator for teaching population genetics. *Bioinform. Adv.* **3**, vbad087 (2023). 37456508 10.1093/bioadv/vbad087 PMC10343943

[9] Traub, R. D. et al. Single-column thalamocortical network model exhibiting gamma oscillations, sleep spindles, and epileptogenic bursts. *J. Neurophysiol.* **93**, 2194–2232 (2005). 15525801 10.1152/jn.00983.2004

[10] D’Angelo, E. et al. Modeling the cerebellar microcircuit: new strategies for a long-standing issue. *Front. Cell Neurosci.* **10**, 176 (2016). 27458345 10.3389/fncel.2016.00176 PMC4937064

[11] Osborne, H. et al. MIIND: a model-agnostic simulator of neural populations. *Front Neuroinform* **15**, 614881 (2021). 34295233 10.3389/fninf.2021.614881 PMC8291130

[12] Tomlinson, I. P. & Bodmer, W. F. Failure of programmed cell death and differentiation as causes of tumors: some simple mathematical models. *Proc. Natl Acad. Sci. USA* **92**, 11130–11134 (1995). 7479951 10.1073/pnas.92.24.11130 PMC40585

[13] Johnston, M. D., Edwards, C. M., Bodmer, W. F., Maini, P. K. & Chapman, S. J. Mathematical modeling of cell population dynamics in the colonic crypt and in colorectal cancer. *Proc. Natl Acad. Sci. USA* **104**, 4008–4013 (2007). 17360468 10.1073/pnas.0611179104 PMC1820699

[14] Höhme, S. et al. Mathematical modelling of liver regeneration after intoxication with CCl(4). *Chem. Biol. Interact.* **168**, 74–93 (2007). 17442287 10.1016/j.cbi.2007.01.010

[15] Hoehme, S. et al. Prediction and validation of cell alignment along microvessels as order principle to restore tissue architecture in liver regeneration. *Proc. Natl Acad. Sci. USA* **107**, 10371–10376 (2010). 20484673 10.1073/pnas.0909374107 PMC2890786

[16] Metzcar, J., Wang, Y., Heiland, R. & Macklin, P. A review of cell-based computational modeling in cancer biology. *JCO Clin. Cancer Inf.* **3**, 1–13 (2019). 10.1200/CCI.18.00069 PMC6584763 30715927

[17] Chamseddine, I. M. & Rejniak, K. A. Hybrid modeling frameworks of tumor development and treatment. *Wiley Interdiscip. Rev. Syst. Biol. Med.* **12**, e1461 (2020). 31313504 10.1002/wsbm.1461 PMC6898741

[18] Bekisz, S. & Geris, L. Cancer modeling: From mechanistic to data-driven approaches, and from fundamental insights to clinical applications. *J. Comput. Sci.* **46**, 101198 (2020).

[19] West, J., Robertson-Tessi, M. & Anderson, A. R. A. Agent-based methods facilitate integrative science in cancer. *Trends Cell Biol.* **33**, 300–311 (2023). 36404257 10.1016/j.tcb.2022.10.006 PMC10918696

[20] Ma, C. & Gurkan-Cavusoglu, E. A comprehensive review of computational cell cycle models in guiding cancer treatment strategies. *NPJ Syst. Biol. Appl* **10**, 71 (2024). 38969664 10.1038/s41540-024-00397-7 PMC11226463

[21] Cogno, N., Axenie, C., Bauer, R. & Vavourakis, V. Agent-based modeling in cancer biomedicine: applications and tools for calibration and validation. *Cancer Biol. Ther.* **25**, 2344600 (2024). 38678381 10.1080/15384047.2024.2344600 PMC11057625

[22] Shmulevich, I., Dougherty, E. R., Kim, S. & Zhang, W. Probabilistic Boolean networks: a rule-based uncertainty model for gene regulatory networks. *Bioinformatics* **18**, 261–274 (2002). 11847074 10.1093/bioinformatics/18.2.261

[23] Albert, I., Thakar, J., Li, S., Zhang, R. & Albert, R. Boolean network simulations for life scientists. *Source Code Biol. Med.* **3**, 16 (2008). 19014577 10.1186/1751-0473-3-16 PMC2603008

[24] Stoll, G. et al. MaBoSS 2.0: an environment for stochastic Boolean modeling. *Bioinformatics* **33**, 2226–2228 (2017). 28881959 10.1093/bioinformatics/btx123

[25] Izaguirre, J. A. et al. CompuCell, a multi-model framework for simulation of morphogenesis. *Bioinformatics* **20**, 1129–1137 (2004). 14764549 10.1093/bioinformatics/bth050

[26] Swat, M. H. et al. Multi-scale modeling of tissues using CompuCell3D. *Methods Cell Biol.* **110**, 325–366 (2012). 22482955 10.1016/B978-0-12-388403-9.00013-8 PMC3612985

[27] Andasari, V. et al. Computational model of wound healing: EGF secreted by fibroblasts promotes delayed re-epithelialization of epithelial keratinocytes. *Integr. Biol. (Camb.)* **10**, 605–634 (2018). 30206629 10.1039/c8ib00048d PMC6571173

[28] Khataee, H., Czirok, A. & Neufeld, Z. Multiscale modelling of motility wave propagation in cell migration. *Sci. Rep.* **10**, 8128 (2020). 32424155 10.1038/s41598-020-63506-6 PMC7235313

[29] Fortuna, I. et al. CompuCell3D simulations reproduce mesenchymal cell migration on flat substrates. *Biophys. J.* **118**, 2801–2815 (2020). 32407685 10.1016/j.bpj.2020.04.024 PMC7264849

[30] Merks, R. M. H. & Glazier, J. A. A cell-centered approach to developmental biology. *Phys. A: Stat. Mech. Appl.* **352**, 113–130 (2005).

[31] Daub, J. T. & Merks, R. M. H. Cell-based computational modeling of vascular morphogenesis using Tissue Simulation Toolkit. *Methods Mol. Biol.* **1214**, 67–127 (2015). 25468600 10.1007/978-1-4939-1462-3_6

[32] Niculescu, I., Textor, J. & de Boer, R. J. Crawling and gliding: a computational model for shape-driven cell migration. *PLoS Comput. Biol.* **11**, e1004280 (2015). 26488304 10.1371/journal.pcbi.1004280 PMC4619082

[33] Tahir, H., Niculescu, I., Bona-Casas, C., Merks, R. M. H. & Hoekstra, A. G. An in silico study on the role of smooth muscle cell migration in neointimal formation after coronary stenting. *J. R. Soc. Interface* **12**, 20150358 (2015). 26063828 10.1098/rsif.2015.0358 PMC4528603

[34] Rens, E. G., Zeegers, M. T., Rabbers, I., Szabó, A. & Merks, R. M. H. Autocrine inhibition of cell motility can drive epithelial branching morphogenesis in the absence of growth. *Philos. Trans. R. Soc. Lond. B: Biol. Sci.* **375**, 20190386 (2020). 32713299 10.1098/rstb.2019.0386 PMC7423378

[35] de Jong, M. A. et al. The shapes of elongating gastruloids are consistent with convergent extension driven by a combination of active cell crawling and differential adhesion. *PLoS Comput. Biol.* **20**, e1011825 (2024). 38306399 10.1371/journal.pcbi.1011825 PMC10866519

[36] Hoehme, S. & Drasdo, D. A cell-based simulation software for multi-cellular systems. *Bioinformatics* **26**, 2641–2642 (2010). 20709692 10.1093/bioinformatics/btq437 PMC2951083

[37] Ghaffarizadeh, A., Heiland, R., Friedman, S. H., Mumenthaler, S. M. & Macklin, P. PhysiCell: An open source physics-based cell simulator for 3-D multicellular systems. *PLoS Comput. Biol.* **14**, e1005991 (2018). 29474446 10.1371/journal.pcbi.1005991 PMC5841829

[38] Heiland, R. et al. PhysiCell Studio: a graphical tool to make agent-based modeling more accessible. *GigaByte* **2024**, gigabyte128 (2024). 10.46471/gigabyte.128 PMC11211762 38948511

[39] Stoll, G., Viara, E., Barillot, E. & Calzone, L. Continuous time Boolean modeling for biological signaling: application of Gillespie algorithm. *BMC Syst. Biol.* **6**, 116 (2012). 22932419 10.1186/1752-0509-6-116 PMC3517402

[40] Stoll, G. et al. UPMaBoSS: a novel framework for dynamic cell population modeling. *Front Mol. Biosci.* **9**, 800152 (2022). 35309516 10.3389/fmolb.2022.800152 PMC8924294

[41] Letort, G. et al. PhysiBoSS: a multi-scale agent-based modelling framework integrating physical dimension and cell signalling. *Bioinformatics* **35**, 1188–1196 (2019). 30169736 10.1093/bioinformatics/bty766 PMC6449758

[42] Ponce-de-Leon, M. et al. PhysiBoSS 2.0: a sustainable integration of stochastic Boolean and agent-based modelling frameworks. *npj Syst. Biol. Appl.* **9**, 1–12 (2023). 37903760 10.1038/s41540-023-00314-4 PMC10616087

[43] Fouliard, S., Benhamida, S., Lenuzza, N. & Xavier, F. Modeling and simulation of cell populations interaction. *Math. Computer Model.* **49**, 2104–2108 (2009).

[44] Altinok, A., Gonze, D., Lévi, F. & Goldbeter, A. An automaton model for the cell cycle. *Interface Focus* **1**, 36–47 (2011). 22419973 10.1098/rsfs.2010.0009 PMC3262245

[45] Taylor, W., Katsimitsoulia, Z. & Poliakov, A. Simulation of cell movement and interaction. *J. Bioinform. Comput Biol.* **9**, 91–110 (2011). 21328708 10.1142/s0219720011005318

[46] Wortel, I. M. N. et al. CelltrackR: An R package for fast and flexible analysis of immune cell migration data. *Immunoinformatics (Amst.)* **1–2**, 100003 (2021). 37034276 10.1016/j.immuno.2021.100003 PMC10079262

[47] Toscano, E., Sepe, L., Del Giudice, G., Tufano, R. & Paolella, G. A three component model for superdiffusive motion effectively describes migration of eukaryotic cells moving freely or under a directional stimulus. *PLoS ONE* **17**, e0272259 (2022). 35917375 10.1371/journal.pone.0272259 PMC9345344

[48] Cooper, S. Control and maintenance of mammalian cell size. *BMC Cell Biol.* **5**, 35 (2004). 15456512 10.1186/1471-2121-5-35 PMC524481

[49] Schmoller, K. M. & Skotheim, J. M. The biosynthetic basis of cell size control. *Trends Cell Biol.* **25**, 793–802 (2015). 26573465 10.1016/j.tcb.2015.10.006 PMC6773270

[50] Cadart, C. et al. Fluorescence eXclusion measurement of volume in live cells. *Methods Cell Biol.* **139**, 103–120 (2017). 28215332 10.1016/bs.mcb.2016.11.009

[51] Cadart, C., Venkova, L., Recho, P., Lagomarsino, M. C. & Piel, M. The physics of cell-size regulation across timescales. *Nat. Phys.* **15**, 993–1004 (2019).

[52] Cadart, C., Venkova, L., Piel, M. & Cosentino Lagomarsino, M. Volume growth in animal cells is cell cycle dependent and shows additive fluctuations. *Elife* **11**, e70816 (2022). 35088713 10.7554/eLife.70816 PMC8798040

[53] van der Bosch, J. Complex population kinetics in 3T3 and SV40-3T3 cell cultures. Involvement of division, inhibition of division and death of cells. *Exp. Cell Res.* **117**, 111–119 (1978). 720397 10.1016/0014-4827(78)90433-0

[54] Kues, W. A. et al. Cell cycle synchronization of porcine fetal fibroblasts: effects of serum deprivation and reversible cell cycle inhibitors. *Biol. Reprod.* **62**, 412–419 (2000). 10642581 10.1095/biolreprod62.2.412

[55] Oki, T. et al. A novel cell-cycle-indicator, mVenus-p27K-, identifies quiescent cells and visualizes G0-G1 transition. *Sci. Rep.* **4**, 4012 (2014). 24500246 10.1038/srep04012 PMC3915272

[56] Pulianmackal, A. J. et al. Monitoring spontaneous quiescence and asynchronous proliferation-quiescence decisions in prostate cancer cells. *Front. Cell Dev. Biol.* **9**, 728663 (2021). 34957090 10.3389/fcell.2021.728663 PMC8703172

[57] Fan, Y. & Meyer, T. Molecular control of cell density-mediated exit to quiescence. *Cell Rep.* **36**, 109436 (2021). 34320337 10.1016/j.celrep.2021.109436 PMC8924979

[58] Insall, R. H., Paschke, P. & Tweedy, L. Steering yourself by the bootstraps: how cells create their own gradients for chemotaxis. *Trends Cell Biol.* **32**, 585–596 (2022). 35351380 10.1016/j.tcb.2022.02.007

[59] Insall, R. H. Receptors, enzymes and self-attraction as autocrine generators and amplifiers of chemotaxis and cell steering. *Curr. Opin. Cell Biol.* **81**, 102169 (2023). 37075582 10.1016/j.ceb.2023.102169

[60] Tweedy, L., Susanto, O. & Insall, R. H. Self-generated chemotactic gradients-cells steering themselves. *Curr. Opin. Cell Biol.* **42**, 46–51 (2016). 27105308 10.1016/j.ceb.2016.04.003

[61] Tweedy, L., Knecht, D. A., Mackay, G. M. & Insall, R. H. Self-generated chemoattractant gradients: attractant depletion extends the range and robustness of chemotaxis. *PLoS Biol.* **14**, e1002404 (2016). 26981861 10.1371/journal.pbio.1002404 PMC4794234

[62] Susanto, O. et al. LPP3 mediates self-generation of chemotactic LPA gradients by melanoma cells. *J. Cell Sci.* **130**, 3455–3466 (2017). 28871044 10.1242/jcs.207514 PMC5665449

[63] Stuelten, C. H. Moving in and out: dispersion of cells in self-generated gradients. *J. Clin. Cell Immunol.* **8**, 507 (2017). 28868205 10.4172/2155-9899.1000507 PMC5578429

[64] Tweedy, L. et al. Seeing around corners: Cells solve mazes and respond at a distance using attractant breakdown. *Science* **369**, eaay9792 (2020). 10.1126/science.aay9792 32855311

[65] Tweedy, L. & Insall, R. H. Self-generated gradients yield exceptionally robust steering cues. *Front Cell Dev. Biol.* **8**, 133 (2020). 32195256 10.3389/fcell.2020.00133 PMC7066204

[66] Paspunurwar, A. S., Moure, A. & Gomez, H. Dynamic cluster field modeling of collective chemotaxis. *Sci. Rep.* **14**, 25162 (2024). 39448677 10.1038/s41598-024-75653-1 PMC11502788

[67] Klepeis, V. E., Weinger, I., Kaczmarek, E. & Trinkaus-Randall, V. P2Y receptors play a critical role in epithelial cell communication and migration. *J. Cell Biochem.* **93**, 1115–1133 (2004). 15449317 10.1002/jcb.20258

[68] Yang, L., Cranson, D. & Trinkaus-Randall, V. Cellular injury induces activation of MAPK via P2Y receptors. *J. Cell Biochem.* **91**, 938–950 (2004). 15034929 10.1002/jcb.10774

[69] Weinger, I., Klepeis, V. E. & Trinkaus-Randall, V. Tri-nucleotide receptors play a critical role in epithelial cell wound repair. *Purinergic Signal* **1**, 281–292 (2005). 18404512 10.1007/s11302-005-8132-6 PMC2096543

[70] Boucher, I., Yang, L., Mayo, C., Klepeis, V. & Trinkaus-Randall, V. Injury and nucleotides induce phosphorylation of epidermal growth factor receptor: MMP and HB-EGF dependent pathway. *Exp. Eye Res.* **85**, 130–141 (2007). 17490650 10.1016/j.exer.2007.03.009 PMC2577227

[71] Yin, J., Xu, K., Zhang, J., Kumar, A. & Yu, F.-S. X. Wound-induced ATP release and EGF receptor activation in epithelial cells. *J. Cell Sci.* **120**, 815–825 (2007). 17284517 10.1242/jcs.03389 PMC1853294

[72] Block, E. R. & Klarlund, J. K. Wounding sheets of epithelial cells activates the epidermal growth factor receptor through distinct short- and long-range mechanisms. *Mol. Biol. Cell* **19**, 4909–4917 (2008). 18799627 10.1091/mbc.E08-01-0097 PMC2575185

[73] Ghilardi, S. J., O’Reilly, B. M. & Sgro, A. E. Intracellular signaling dynamics and their role in coordinating tissue repair. *Wiley Interdiscip. Rev. Syst. Biol. Med* **12**, e1479 (2020). 32035001 10.1002/wsbm.1479 PMC7187325

[74] Altrock, P. M., Liu, L. L. & Michor, F. The mathematics of cancer: integrating quantitative models. *Nat. Rev. Cancer* **15**, 730–745 (2015). 26597528 10.1038/nrc4029

[75] Jarrett, A. M. et al. Mathematical models of tumor cell proliferation: a review of the literature. *Expert Rev. Anticancer Ther.* **18**, 1271–1286 (2018). 30252552 10.1080/14737140.2018.1527689 PMC6295418

[76] Engeland, K. Cell cycle regulation: p53-p21-RB signaling. *Cell Death Differ.* **29**, 946–960 (2022). 35361964 10.1038/s41418-022-00988-z PMC9090780

[77] Catapano, R. et al. Biological relevance of ZNF224 expression in chronic lymphocytic leukemia and its implication IN NF-kB pathway regulation. *Front. Mol. Biosci.* **9**, 1010984 (2022). 36425656 10.3389/fmolb.2022.1010984 PMC9681601

[78] Guo, Q. et al. NF-κB in biology and targeted therapy: new insights and translational implications. *Signal Transduct. Target Ther.* **9**, 53 (2024). 38433280 10.1038/s41392-024-01757-9 PMC10910037

[79] Pellarin, I. et al. Cyclin-dependent protein kinases and cell cycle regulation in biology and disease. *Signal Transduct. Target Ther.* **10**, 11 (2025). 39800748 10.1038/s41392-024-02080-z PMC11734941

[80] Todaro, G. J. & Green, H. Quantitative studies of the growth of mouse embryo cells in culture and their development into established lines. *J. Cell Biol.* **17**, 299–313 (1963). 13985244 10.1083/jcb.17.2.299 PMC2106200

[81] Sepe, L., Ferrari, M. C., Cantarella, C., Fioretti, F. & Paolella, G. Ras activated ERK and PI3K pathways differentially affect directional movement of cultured fibroblasts. *Cell Physiol. Biochem.* **31**, 123–142 (2013). 23363700 10.1159/000343355

[82] GEY, G. Tissue culture studies of the proliferative capacity of cervical carcinoma and normal epithelium. *Cancer Res.* **12**, 264–265 (1952).

[83] Bubeník, J. et al. Established cell line of urinary bladder carcinoma (T24) containing tumour-specific antigen. *Int J. Cancer* **11**, 765–773 (1973). 4133950 10.1002/ijc.2910110327

[84] Cailleau, R., Young, R., Olivé, M. & Reeves, W. J. Breast tumor cell lines from pleural effusions. *J. Natl Cancer Inst.* **53**, 661–674 (1974). 4412247 10.1093/jnci/53.3.661 PMC7364228

[85] Todaro, G. J., Fryling, C. & De Larco, J. E. Transforming growth factors produced by certain human tumor cells: polypeptides that interact with epidermal growth factor receptors. *Proc. Natl Acad. Sci. USA* **77**, 5258–5262 (1980). 6254071 10.1073/pnas.77.9.5258 PMC350037

[86] Giard, D. J. et al. In vitro cultivation of human tumors: establishment of cell lines derived from a series of solid tumors. *J. Natl Cancer Inst.* **51**, 1417–1423 (1973). 4357758 10.1093/jnci/51.5.1417

[87] Herlyn, M. et al. Primary melanoma cells of the vertical growth phase: similarities to metastatic cells. *J. Natl Cancer Inst.* **74**, 283–289 (1985). 3856042

[88] Kaighn, M. E., Lechner, J. F., Narayan, K. S. & Jones, L. W. Prostate carcinoma: tissue culture cell lines. *Natl Cancer Inst. Monogr.* **49**, 17–21 (1978). 571045

[89] Fogh, J., Fogh, J. M. & Orfeo, T. One hundred and twenty-seven cultured human tumor cell lines producing tumors in nude mice. *J. Natl Cancer Inst.* **59**, 221–226 (1977). 327080 10.1093/jnci/59.1.221

[90] Cantarella, C., Sepe, L., Fioretti, F., Ferrari, M. C. & Paolella, G. Analysis and modelling of motility of cell populations with MotoCell. *BMC Bioinform.* **10**, S12 (2009). 10.1186/1471-2105-10-S12-S12 PMC2762061 19828072

[91] Fürth, R. Die Brownsche Bewegung bei Berücksichtigung einer Persistenz der Bewegungsrichtung. Mit Anwendungen auf die Bewegung lebender Infusorien. *Z. Phys.* **2**, 244–256 (1920).

[92] Alt, W. Correlation Analysis of Two-Dimensional Locomotion Paths. In: *Biological Motion*: *Proceedings of a Workshop held in Königswinter, Germany, March 16–19, 1989* (eds. Alt, W. & Hoffmann, G.) 254–268 (Springer, Berlin, Heidelberg, 1990). 10.1007/978-3-642-51664-1_18.

