# Supplementary material for: Cell populations simulated in silico within SimulCell accurately reproduce the behaviour of experimental cell cultures
Source: NPJ Syst Biol Appl. 2025 May 17;11:48. doi: 10.1038/s41540-025-00518-w (PMC12084383; doi:10.1038/s41540-025-00518-w)
Supplement: Supplementary file 1 — Supplementary Information [file 41540_2025_518_MOESM1_ESM.pdf]

Supplementary Information

Supplementary Table 1. Proliferation parameters obtained from simulated cell populations

sim. time (h)	n start cells	seeding area (µm)	constrain to seeding area	requested repl. time (h)	calculated repl. time (h)	n final cells	population dupl. time (h)	n dead	G_0 fraction (avg)
48	100	1600x1200	no	20	20.4	395	24.2	39	0.03
48	100	1600x1200	no	24	24.6	225	41.0	60	0.04
48	100	1600x1200	no	28	27.5	216	43.2	55	0.07
48	100	1600x1200	no	32	31.7	193	50.6	61	0.05
48	100	800x600	no	20	20.9	421	23.1	24	0.10
48	100	800x600	no	24	24.9	343	27.0	22	0.09
48	100	800x600	no	28	28.2	298	30.5	16	0.07
48	100	800x600	no	32	32.7	244	37.3	22	0.10
48	100	800x600	yes	20	21.0	385	24.7	39	0.17
48	100	800x600	yes	24	24.8	351	26.5	16	0.13
48	100	800x600	yes	28	28.5	289	31.4	18	0.15
48	100	800x600	yes	32	32.2	235	38.9	26	0.15

Four cell populations, with replication time ranging between 20 and 32 hours, were simulated while freely growing on a large plate surface (1600µmx1200µm) and a smaller one (800µmx600µm) in absence and presence of a physical constraint limiting cell movement. Calculated replication time, measured as average cell age at mitosis, the number of final cell, population duplication time, the number of dead cells and fraction of cells in G_0 phase are reported for each simulated population.

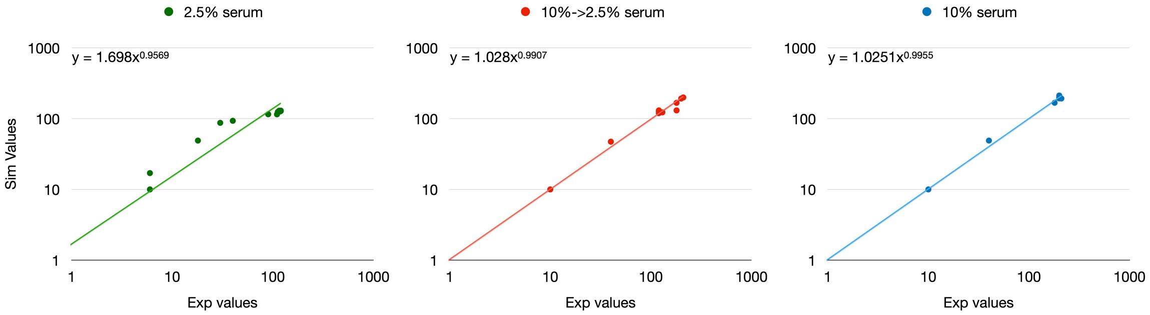

Supplementary Figure 1. Growth curve validation. For simulated populations of Fig. 2a, the number of cells was compared with the number of cells of the corresponding experimental population[1]. Cell numbers are expressed as cells per unit surface (0.58 mm^2). For each comparison, the plotted trend line is calculated using the function reported in the top-left corner.

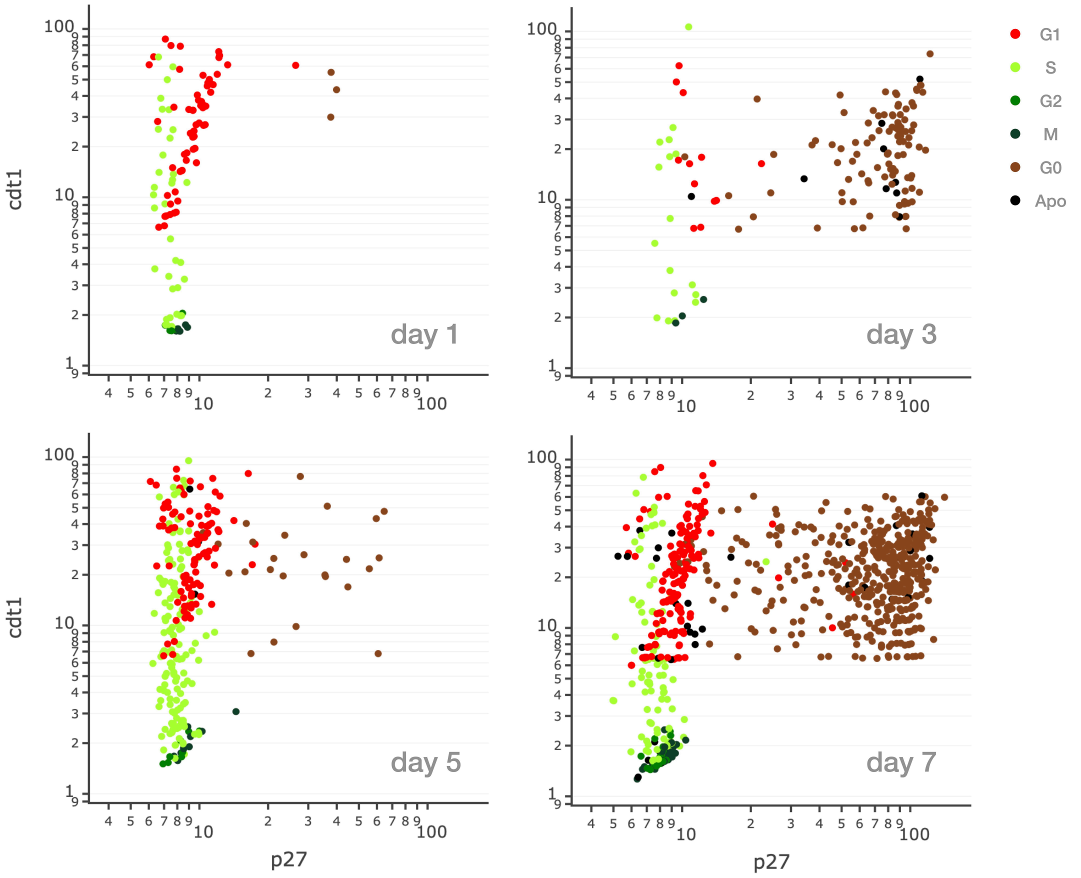

Supplementary Figure 2. Analysis of serum starvation and rescue of simulated populations by following the quantity of p27/cdt1 and cell cycle phase. Snapshots of the same simulated population as in Fig. 2e and f at day 1, 3, 5 and 7 are reported by plotting each cell according to cdt1 and p27 concentration and by using a color code for distinguishing the cycle phase of each cell .

Supplementary Table 2. Migration parameters determined for different cell populations.

tested population	cell line	tissue origin	avg disp (µm) per 40’	rand module (µm)	pers module (µm)	bias module (µm)	pers /rand module	bias / ran module
1	HeLa	cervical cancer	10.13	10.08	2.55 ± 0.70	0.48 ± 0.53	0.253	0.048
1w			10.56	8.29	2.17 ± 0.91	5.45 ± 0.73	0.262	0.657
2	T24	bladder carcinoma	17.18	14.60	10.43 ± 0.87	1.18 ± 0.63	0.714	0.081
2w			9.73	7.43	3.32 ± 0.73	4.31 ± 0.59	0.447	0.580
3	MDA-MB-231	metastatic breast cancer	7.65	7.18	2.47 ± 0.45	0.39 ± 0.31	0.344	0.054
4	PC3	prostatic adenocarcinoma	13.04	13.22	0.37 ± 0.96	0.04 ± 0.69	0.028	0.003
5	A2058	melanoma	11.15	10.99	5.19 ± 0.77	0.83 ± 0.54	0.472	0.076
6	A375	melanoma	4.57	4.47	1.10 ± 0.32	0.18 ± 0.24	0.246	0.040
7	Calu	lung adenocarcinoma	4.42	4.15	0.01 ± 0.41	0.14 ± 0.33	0.002	0.034
8	wm115	melanoma	8.14	8.75	2.65 ± 0.89	0.11 ± 0.65	0.303	0.013
9	NIH-3T3	mouse embryo fibroblasts	9.68	9.03	2.72 ± 0.55	0.57 ± 0.40	0.301	0.063
9w			9.34	6.89	1.42 ± 0.76	6.36 ± 0.63	0.206	0.923
10	NIH-Ras	mouse embryo fibroblasts	16.35	14.93	9.35 ± 0.81	1.09 ± 0.57	0.626	0.073

HeLa, T24**,** MDA-MB-231, PC3, A2058, A375, Calu, wm115, NIH-3T3 and NIH-Ras cells were grown under standard culture conditions and motion parameters were calculated for a 12 hour time window. Average displacement, random, persistence and bias modules as well as persistence and bias modules normalized against the corresponding random module are reported in each column. For HeLa, T24 and NIH-3T3 cells the same parameters were also calculated for a subpopulation of cells present at the front of a wound (rows 1w, 2w and 9w).

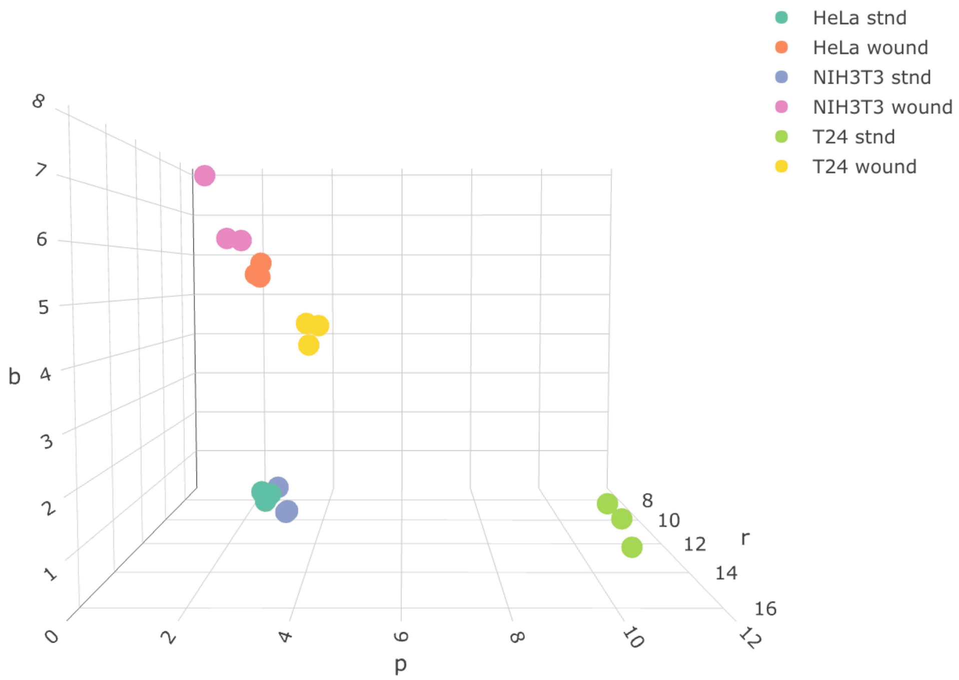

Supplementary Figure 3. Simulated cell populations show values for random, persistence and bias very similar to those of the corresponding experimental populations. The same experimental and simulated populations as in Table1 are positioned in 3D space according to their values of random, persistence and bias components.

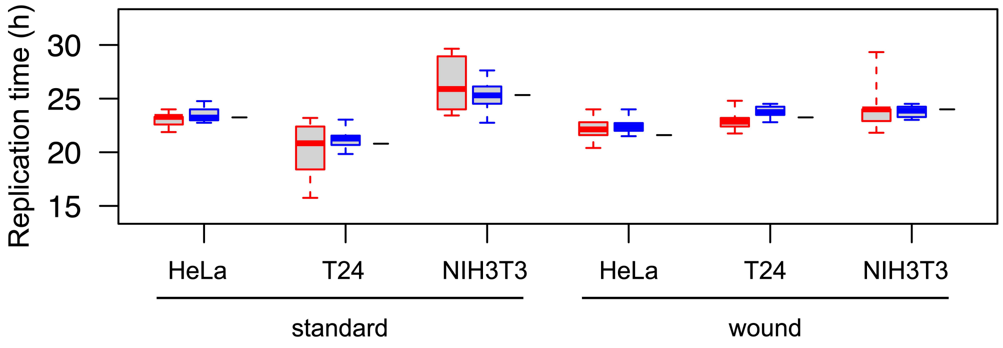

Supplementary Figure 4. The distributions of the replication time of simulated cell populations are tight and show values very similar to those of the corresponding experimental cultures. For both standard and wound conditions, the distributions of the replication time of the same populations as in Fig. 4c are reported. For each cell type in each growth condition, red boxes are used to report the distributions of replication times obtained by 10 populations simulated starting with the same number of cells as in the corresponding experimental one, the blue ones, instead, indicate the results for other 10 populations with a standard number of 50 starting cells, black dashes indicate the value obtained from the experimental populations.

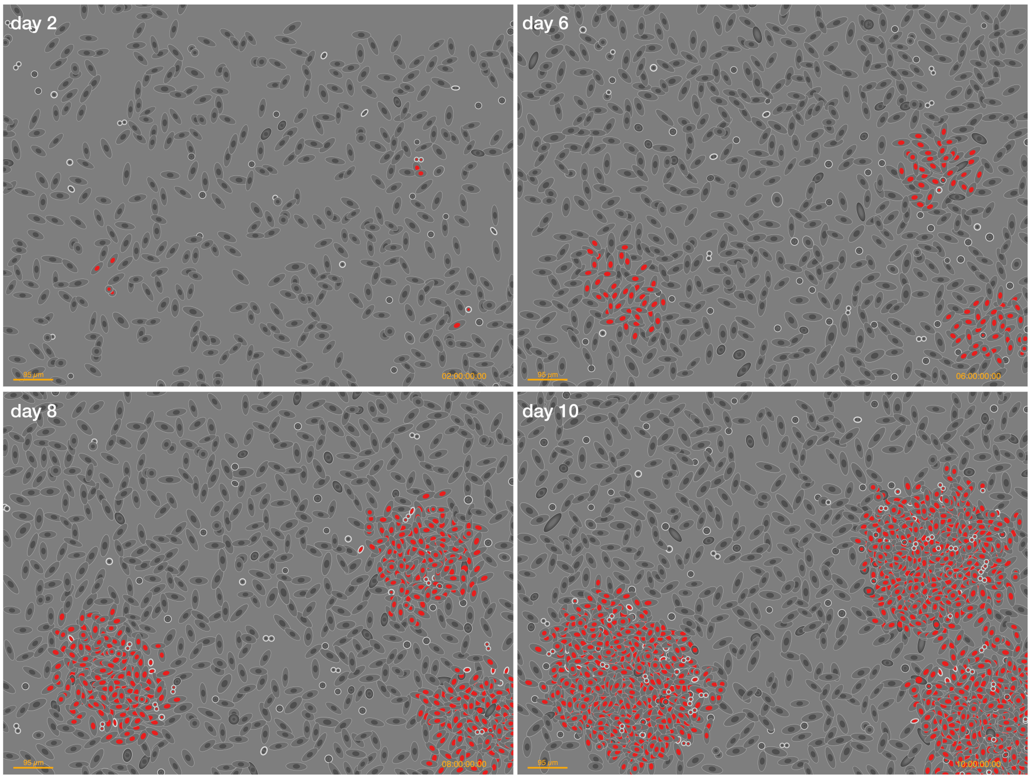

Supplementary Figure 5. Simulated NIH3T3-like cells modified by acquiring a constitutively active Ras gene by transfection. The images represent the same population as in Fig. 8e-g, with, highlighted in red, the cells, which have acquired the constitutively active Ras gene.

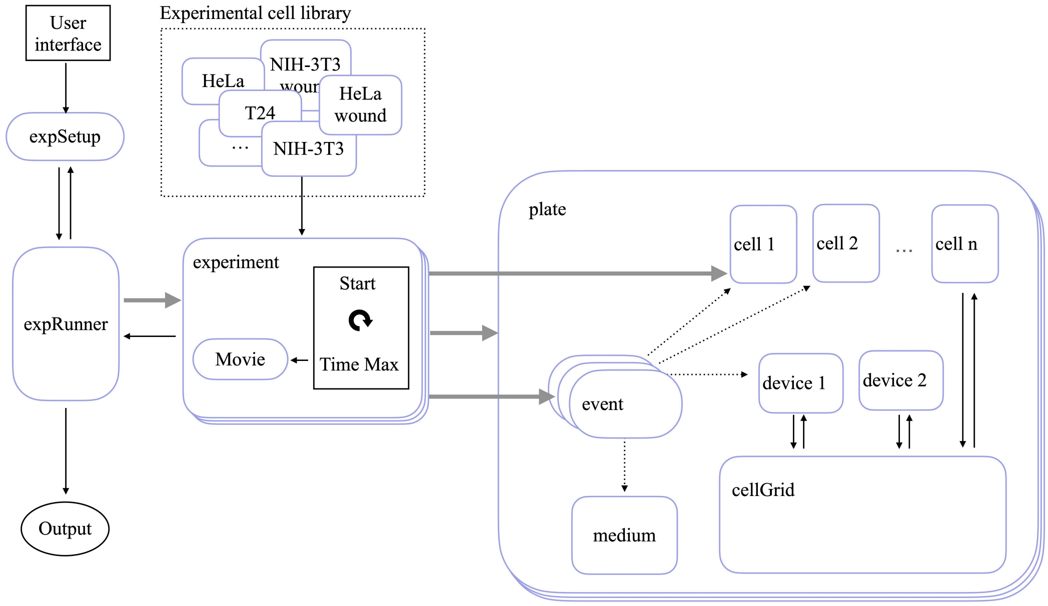

Supplementary Figure 6. Schematic representation of the simulator architecture. In the representation of *SimulCell* architecture rounded boxes correspond to the different objects on which the simulator is based while arrows specify relations and data flow between objects. Specifically, the continue arrows indicate transfer of data, the thick grey arrows indicate object creation and the dotted arrows indicate that the object modifies features and/or behaviour of another one.

Supplementary Table 3. Performance parameters obtained from simulated populations.

simulation time (days)	n start cells	seeding area (µm)	n final cells	execution time (secs)	Secs per cell per day	cell updates per second
1	100	2000 x1500	167	7	0.05	1901
2	100	2000 x1500	275	17	0.05	1767
3	100	2000 x1500	422	32	0.04	1705
4	100	2000 x1500	815	69	0.04	1630
5	100	2000 x1500	1622	166	0.04	1550
3	50	1500 x1000	243	17	0.04	1757
3	100	2000 x1500	482	36	0.04	1679
3	200	3000 x2000	943	78	0.05	1589
3	500	5000 x3000	2377	249	0.06	1393

Simulation performance was evaluated by analyzing typical culture conditions, including different start cell numbers (50-500 cells) and simulation durations (1-5 days). Number of final cells, execution time, seconds per cell per day and cell updates per second are reported for each simulated population.

Supplementary Movie1. Formation of antibiotic resistant clones. The movie reports the formation of antibiotic resistant clones after DNA transfection of NIH3T3-like population. Cell visualisation uses the "symbolic" representation of Fig7c.

Supplementary Movie2. Production of transformed foci. The movie reports the production of foci by overgrowth after transformation by exogenous DNA within a NIH3T3-like population. Cell visualisation uses the "symbolic" representation of Fig7c.

Supplementary Movie3. Lysis plaques formation after viral inflection. The movie reports simulated VeroE6 cells infected by a lytic virus. Cell visualisation uses the "symbolic" representation of Fig7c.

References

1. van der Bosch, J. Complex population kinetics in 3T3 and SV40-3T3 cell cultures. Involvement of division, inhibition of division and death of cells. *Exp Cell Res* **117**, 111–119 (1978).
